# Barcoding against a paradox? Combined molecular species delineations reveal multiple cryptic lineages in elusive meiofaunal sea slugs

**DOI:** 10.1186/1471-2148-12-245

**Published:** 2012-12-18

**Authors:** Katharina M Jörger, Jon L Norenburg, Nerida G Wilson, Michael Schrödl

**Affiliations:** 1 Mollusca Department, Bavarian State Collection of Zoology, Münchhausenstr.21, 81247, München, Germany; 2 Department Biology II, Ludwig-Maximilians-Universität, BioZentrum Martinsried, Großhadernerstr. 2, 82152, Planegg-Martinsried, Germany; 3 Department of Invertebrate Zoology, National Museum of Natural History, Smithsonian Institution, Washington, DC, 20560, USA; 4 The Australian Museum, 6 College Street, Sydney, NSW, 2010, Australia

## Abstract

**Background:**

Many marine meiofaunal species are reported to have wide distributions, which creates a paradox considering their hypothesized low dispersal abilities. Correlated with this paradox is an especially high taxonomic deficit for meiofauna, partly related to a lower taxonomic effort and partly to a high number of putative cryptic species. Molecular-based species delineation and barcoding approaches have been advocated for meiofaunal biodiversity assessments to speed up description processes and uncover cryptic lineages. However, these approaches show sensitivity to sampling coverage (taxonomic and geographic) and the success rate has never been explored on mesopsammic Mollusca.

**Results:**

We collected the meiofaunal sea-slug *Pontohedyle* (Acochlidia, Heterobranchia) from 28 localities worldwide. With a traditional morphological approach, all specimens fall into two morphospecies. However, with a multi-marker genetic approach, we reveal multiple lineages that are reciprocally monophyletic on single and concatenated gene trees in phylogenetic analyses. These lineages are largely concordant with geographical and oceanographic parameters, leading to our primary species hypothesis (PSH). In parallel, we apply four independent methods of molecular based species delineation: General Mixed Yule Coalescent model (GMYC), statistical parsimony, Bayesian Species Delineation (BPP) and Automatic Barcode Gap Discovery (ABGD). The secondary species hypothesis (SSH) is gained by relying only on uncontradicted results of the different approaches (‘minimum consensus approach’), resulting in the discovery of a radiation of (at least) 12 mainly cryptic species, 9 of them new to science, some sympatric and some allopatric with respect to ocean boundaries. However, the meiofaunal paradox still persists in some *Pontohedyle* species identified here with wide coastal and trans-archipelago distributions.

**Conclusions:**

Our study confirms extensive, morphologically cryptic diversity among meiofauna and accentuates the taxonomic deficit that characterizes meiofauna research. We observe for *Pontohedyle* slugs a high degree of morphological simplicity and uniformity, which we expect might be a general rule for meiofauna. To tackle cryptic diversity in little explored and hard-to-sample invertebrate taxa, at present, a combined approach seems most promising, such as multi-marker-barcoding (i.e., molecular systematics using mitochondrial and nuclear markers and the criterion of reciprocal monophyly) combined with a minimum consensus approach across independent methods of molecular species delineation to define candidate species.

## Background

Sediment-associated marine meiofaunal organisms inhabit one of the largest ecosystems on earth – sediment-covered ocean floors and beaches – and comprise a major part of marine biodiversity [1]. However, only a small fraction of the predicted species richness currently is known to science [1-4] and recent surveys have shown a high number of new, undescribed species even in well-studied areas (see [4]). Minute body sizes often prohibit direct visual identification in the field; instead, morphological identification generally requires time-consuming, technologically sophisticated anatomical studies. Additionally, taxonomy frequently is complicated by morphological convergence and/or pronounced intraspecific variation (e.g., [3,5]). In Acochlidia, the most diverse group of meiofaunal slugs, the Microhedylacea, shows ‘regressive evolution’ [6], exhibiting highly simplified organ systems and little morphological diversity even at higher taxonomic levels [7]. Thus, it is challenging to use only morphology to delimit species boundaries in meiofaunal slugs. In consequence of the fragmentary knowledge of meiofaunal taxonomy, this fauna is frequently neglected in conservation and biogeography, and ecological analyses remain superficial despite the undoubted importance of meiofauna; e.g., in the food chain [8].

For taxon-specific analyses, DNA-barcoding has been advocated as a fast and efficient way to reduce the taxonomic deficit and automate taxon determination for ecological research [3,5,8]. DNA-barcoding in its simple, similarity-based form of species identification [9] is not predictive; it fails if no identical sequence has yet been determined and deposited in a voucher database, or if no limit in species boundaries has been established [10,11]. In well-known taxa with good sampling coverage, identification rates via DNA-barcoding can be quite successful (e.g.,[12,13]), but in case of meiofauna finding identical sequences in public databases for a newly collected mollusk or other under-investigated taxon is not expected to become the rule for decades to come. The application of the typical barcoding approach for species delineation - COI in conjunction with a comparison of pairwise distances - in Mollusca has resulted in mixed reports: although the identification success with known taxa was generally high (e.g., [12,14,15]), the determination of a ‘barcoding gap’ (i.e., significant difference between inter- and intraspecific variation) and thus a delimiting threshold has been problematic, especially above local-scale approaches and in undersampled phylogenies [12]. Doubts have also arisen concerning species identification and delimitation based on single-locus DNA sequences, which frequently result in problematic under- or overestimation of species [16-18]. Mitochondrial markers, especially, came under criticism due to possible inadvertent inclusion of nuclear mitochondrial pseudogenes (= nonfunctional copies of mtDNA in the nucleus, or numts) [19], and other mitochondria-specific pitfalls such as reduced effective population size or inconsistent recombination [20]. The risk of incorrect species delineation due to incomplete lineage sorting or introgression can be reduced by analyzing independent loci [21], which is generally considered superior to single-gene approaches [22]. We chose a barcoding approach based on three molecular markers that have been demonstrated to provide good resolution for species delineation in some Mollusca [23-25]. We included, in addition to mitochondrial Cytochrome *c* Oxidase subunit I (COI) and 16S rRNA, nuclear 28S rRNA (large ribosomal subunit – LSU), which has performed well for species separation and was suggested as a signature gene fragment for a DNA taxonomy system for meiobenthos [8,26].

Any method of species delineation is sensitive to sampling [27], and rarity is almost universal when dealing with invertebrates [28]. Rarity is not only a theoretical problem in species delineation methods, but hinders assessment of genetic variability [16,28]; if populations with intermediate haplotype composition are left unsampled, barcoding and molecular-based species delineation approaches tend to overestimate species [18,27]. With large parts of the worldwide meiofauna still unexplored, and patchy, discrete distributions being characteristic for meiofaunal taxa [29], the present-day knowledge of this fauna is prone to incomplete sampling. The rapidly spreading biodiversity crisis with the destruction of habitats and high extinction rates calls for quick surveys and realistic data for efficient conservation strategies (e.g., [16], and references therein). Currently, most molecular species delineation approaches have been conducted on large datasets with dense sampling coverage or on local scales (e.g., [30-35]), with few exceptions using small datasets in integrative approaches (e.g., [36]). Barcoding approaches using COI trees for defining species clusters and revealing gaps between intra- and intercluster distance; multi-locus tree-based methods with or without using diagnostic characters; and a couple of newly developed, tree-independent methods for species delineation, all serve as methods for DNA taxonomy. However, as a solution to address the challenges of the taxonomic impediment in problematic taxa the power of these methods is still largely untested.

Here, we performed thorough phylogenetic analyses of all three molecular markers and integrate available additional data from morphology and geography. In parallel, we applied four different methods of species delineation: 1) The General Mixed Yule Coalescent model (GMYC) [32,33] is a maximum likelihood approach, able to discriminate between population and speciation patterns on a given ultrametric tree; 2) Statistical parsimony [37] is designed to present intraspecific relationships in haplotype networks, but can also be reversed and used to detect species boundaries [33]; 3) Bayesian Species Delineation (BPP) is a method which accounts for uncertainties in gene trees and is promoted as especially useful for delineation of cryptic species in sympatry [22,38]; 4) Automatic Barcoding Gap Discovery (ABGD) [39] is an exploratory tool based on pairwise distances to detect automatically significant difference in intra- and interspecific variation (i.e., barcoding gap), without an *a priori* species hypothesis. Results are compared to a simple single-gene COI barcoding approach in conjunction with pairwise distances.

Our study organism, *Pontohedyle*, is a morphologically well-defined genus of meiofaunal slugs (Acochlidia, Heterobranchia) with two valid species: the well described and abundant *P. milaschewitchii* (Kowalevsky, 1901) from the Black Sea and Mediterranean [40] and the poorly known Western Pacific *P. verrucosa* (Challis, 1970) from the Solomon Islands. In absence of distinguishing morphological characters Jörger *et al.*[41] synonymized the tropical Western Atlantic *‘P. brasilensis’* with its temperate congener *P. milaschewitchii*. This resulted in the only meiofaunal slug with amphi-Atlantic distribution, and the authors pointed out the need to subsequently test this morphological hypothesis with molecular markers to detect possible cryptic species [41]. Sampling efforts in the course of this study revealed a worldwide distribution of the genus. In applying traditional morphological characters for species delineation (external morphology, radula and spicules) all collected material resolved into two morphospecies represented by the currently recognized species (see further details in discussion on species delineation in *Pontohedyle*). Wide-range distributions, as e.g., in *P. milaschewitchii*, are commonly reported for other meiofauna as well, but are treated with suspicion and known as the ‘meiofauna paradox’[42]: Most meiofauna have low reproductive output and lack recognized dispersal stages, such as planktonic larvae [43,44]. Thus, their dispersal abilities and levels of gene flow are assumed to be low [45]. Recent molecular and advanced morphological approaches have revealed putative amphi-Atlantic or even cosmopolitan meiofaunal taxa to be radiations of cryptic species (e.g., [46-52]). Uncovering putative cryptic lineages is fundamental not only for our advances in understanding speciation processes in meiofaunal taxa, but also to understanding historical biogeography.

We present the first species-level phylogenetic analysis in meiofaunal Mollusca to have a world-wide sampling, and aim to 1) establish a workflow of molecular species delineation in rare (or rarely sampled) taxa; 2) test for the presence of putative cryptic species by applying several independent approaches of molecular-based species delineations; 3) test putative wide-range distributions in a meiofaunal slug; and 4) explore the origin and diversification of *Pontohedyle*. Resulting insights into allopatric and sympatric speciation, morphological stasis and distribution are discussed for a better understanding of meiofaunal biogeography and evolution.

## Results

### Phylogenetic analyses and primary species hypotheses

**Figure 1:**
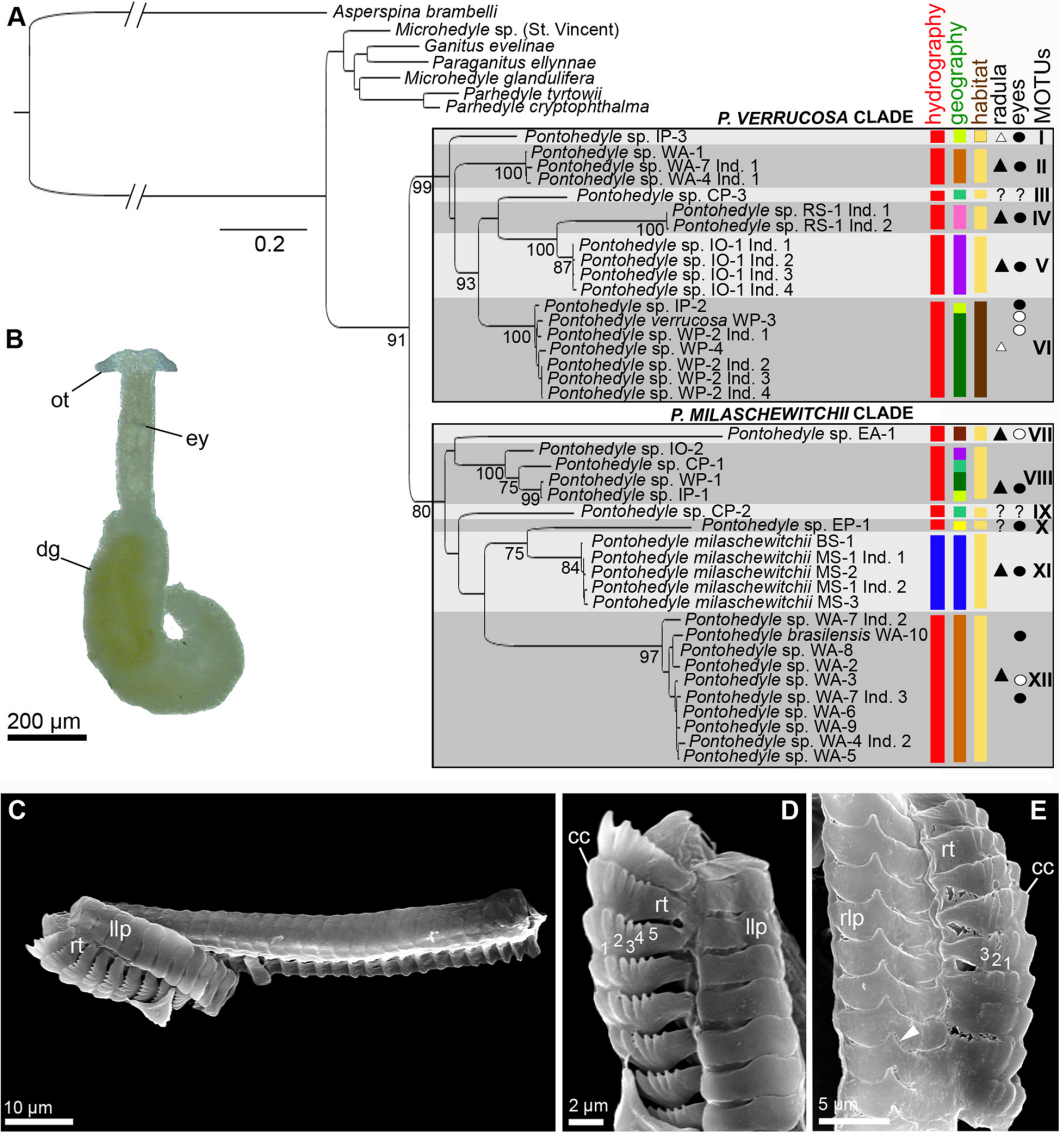
**Molecular phylogeny of *Pontohedyle *with morphological and ecological data plotted.A**. Maximum-likelihood tree of the genus *Pontohedyle* generated with RAxML for the concatenated dataset (28S rRNA, 16S rRNA, COI) analyzed in three partitions. Bootstrap support (BS) above 75 given below nodes (BS within MOTUs shown only for VIII). · eyes externally visible (as in Figure 1B); ○ eyes not visible externally; ▴ lateral radula tooth with denticle (see Figure 1E); Δ lateral radula tooth without denticle (see Figure 1C,D); Hydrography: red = tropical, blue = temperate. Geography: East-Pacific = yellow, Central Indo-Pacific = light-green, Central-Pacific = turquoise, West-Pacific = dark green, Mediterranean and Black Sea = blue, Red Sea = pink, Indian Ocean = purple, West Atlantic = dark brown, East Atlantic = light brown, Habitat: intertidal = brown, subtidal = beige. **B**. Living *Pontohedyle milaschewitchii*. **C**.-**E**. Scanning electron microscopy of *Pontohedyle* radulae, arrowhead indicates denticle in lateral plate of radula, numbers mark lateral cusps of rachidian tooth. **C**.- **D**. Radula of *P. verrucosa*. **E**. Radula of *P. milaschewitchii*. cc = central cusp of rachidian tooth, dg = digestive gland, ey = eyes, llp = left lateral plate, ot = oral tentacles, rlp = right lateral plate, rt = rachidian tooth.

**Figure 2:**
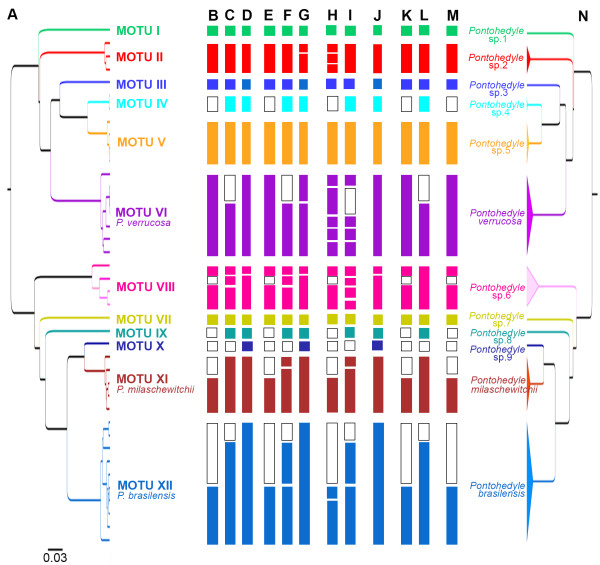
**Molecular based species delineation of the genus *Pontohedyle.*A**. Ultrametric tree generated with BEAST from the concatenated three-marker dataset, with the PSH derived from phylogeny coded by color. **B**-**D**. GMYC single threshold analyses: **B**. COI. **C**. 16S rRNA. **D**. concatenated three-marker dataset. **E**-**G**. GMYC multiple threshold analyses: **E**. COI. **F**. 16S rRNA. **G**. concatenated three-marker dataset. **H**-**I**. Statistical parsimony haplotype networks analyzed with TCS under the 95% parsimony criterion. **H**. COI. **I**. 16S rRNA. **J**. Summary of the Bayesian Species delineation approach, recognizing entities with posterior probability values ≥ 0.95. **K**-**L**. ABGD analyses. **K**. COI. **L**. 16S rRNA. **M**. Fixed delineating pairwise-distance threshold of 11%. **N**. Candidate species (secondary species hypothesis – SSH) under a minimum consensus approach across methods. (Empty squares represent missing data.).

We used a phylogenetic approach to determine molecular operational taxonomic units (MOTUs), i.e. preliminary molecular units unaffected by existing nomenclature serving as a starting point for further species delineation approaches. Our phylogenetic analyses resulted in a stable topology with only minor changes among different analyses with individual or concatenated markers, revealing a complex picture of diversification in *Pontohedyle*. The topology of the maximum-likelihood analyses of the concatenated three-marker dataset analyzed in three partitions is shown in Figure 1A. This topology was quite stable regardless of the partitioning scheme of the dataset or the phylogenetic approach chosen (likelihood, parsimony or bayesian analysis) (see Figures 1A, 2A and Additional file 1). Differences in the topology referred to poorly supported sister group relationships, frequently involving singletons (e.g. MOTUs VII or X).

The genus *Pontohedyle* was monophyletic with high bootstrap support (BS 91; BS values derived from concatenated three-marker ML analyses, see Figure 1A). It was divided in two sister clades, one included *P. verrucosa* from the type locality (BS 99) and the other *P. milaschewitchii* from the type locality (BS 80, see 1A). Both major clades consisted of six lineages each and represented a geographical mixture across ocean boundaries (see Figure 1A). However, relationships among lineages within the major clades were not supported (i.e. BS <50) in many cases.

Distinguishing features traditionally used for morphological species delineation and ecological traits such as hydrographic conditions, geography and habitat were plotted onto the phylogeny (see Figure 1A). Specimens of *Pontohedyle* are externally uniform and easily distinguishable from other acochlids by the lack of rhinophores and the bow-shaped oral tentacles (Figure 1B). No diagnostic differences in external morphology or spicules could be detected between the collected populations apart from eyes externally visible or not (see Figure 1A). Comparative SEM-examination of the available radulae revealed two types of the typically hook-shaped radula (Figure 1C): a lateral tooth without a denticle (*P. verrucosa*, Figure 1D) or with a denticle (*P. milaschewitchii*, Figure 1E).

We identified our MOTUs according to the criterion of reciprocal monophyly across different phylogenetic approaches and between single gene trees and concatenated datasets (partially missing data however resulted in a lack of some MOTUs in single gene trees, see Additional file 1B-D). A combination of the plotted morphological and ecological data led to diagnosable entities. We detected seven terminal clades, which are reciprocally monophyletic with moderate (BS > 75) to strong support (BS > 95) (see Figure 1A and Additional file 1), and also showed short intra- vs. longer interspecific branch lengths. Additionally, 5 singletons were identified as MOTUs based on similar relative branch lengths when compared to the reciprocally monophyletic entities. Parsimony analysis conducted with PAUP v. 4.10 showed lower resolution among clades, which results in MOTU X and XI collapsing to form a single clade and MOTU VII being recovered outside *Pontohedyle* both are considered an artifact, due to long branch attraction and/or respectively missing data (see Additional file 1A). Monophyly and relative branch lengths of the identified MOTUs were unaffected by masking ambiguous parts of the 16S and 28S rRNA alignment. Our phylogenetic analyses in combination with the plotted morphological and ecological data led to a primary species hypothesis, which was subjected to the following species delineation approaches.

### Molecular-based species delineation and secondary species hypotheses

#### Maximum-likelihood (GMYC)

Discriminating between population and speciation patterns, under single-threshold analyses, GMYC identified all MOTUs as separate species, regardless whether based on COI (Figure 2B), 16S rRNA (Figure 2C) or the concatenated three-marker dataset including nuclear 28S rRNA (Figure 2D). Additionally, MOTU VIII was divided into two species (incomplete COI dataset and concatenated dataset), or three species (16S rRNA). In multiple-threshold analyses (Figure 2E-G), GMYC based on 16S rRNA further divided *P. milaschewitchii* (MOTU XI) and *P. brasilensis* (MOTU XII) into two species each (Figure 2F). In the multiple-threshold GMYC-analyses of the concatenated dataset these MOTUs formed one entity, but *P. verrucosa* and MOTU II were divided in two species each (Figure 2G).

#### Statistical parsimony

Haplotype networks were generated via statistical parsimony implemented in TCS 1.21. With the 95% parsimony criterion (default setting) applied to the single-marker alignments of the mitochondrial datasets, TCS generated 17 networks on COI and 19 unconnected haplotype networks based on 16S rRNA (Figure 2H, I). Statistical parsimony was in agreement with our PSH described above and recovered all the identified MOTUs as unconnected networks. Additionally, 16S rRNA analysis split populations identified above as *P. milaschewitchii* and *P. verrucosa* into unconnected haplotypes (Figure 2I). In COI analyses *P. milaschewitchii* formed one entity but populations of *P. verrucosa* showed unconnected networks (Figure 2H). COI results also showed MOTU II and XII (*P. brasilensis*) each including multiple unconnected networks and the ambiguous MOTU VIII (recovered as two or three putative species in GMYC) resulted in two (COI), or four (16S) unconnected haplotypes under statistical parsimony. The nuclear 28S rRNA haplotype network resulted in connected haplotype networks for representatives of two different (morphologically well-supported) outgroup genera (*Microhedyle* and *Paraganitus*). We thus considered this approach problematic for species delineation in *Pontohedyle* and excluded it from our consensus approach.

#### Bayesian species delineation (BPP)

**Figure 3:**
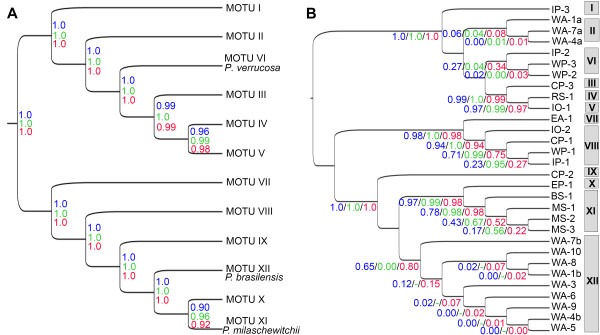
**Bayesian species delimitation for *Pontohedyle. *A**. Results assuming our primary species hypothesis guide tree (12 MOTUs). **B**. Results assuming a guide tree, in which each population is treated as separate species (30 populations – MOTUs are indicated at the left side of the graph). Speciation probability limits are provided for each node under different prior estimates on ancestral population size (θ) and divergence times (τ): 1) prior means 0.1 (blue), 2) prior mean θ = 0.001, τ = 0. 1 (green), 3) prior mean θ = 0.1, τ = 0.001 (red), 4) prior means 0.001 (black). Posterior probabilities are calculated as mean values from repeated analyses. We applied different algorithms and starting seeds, as recommended in the BP&P manual [38].

We ran two sets of Bayesian species delineation analyses: 1) testing the support of the MOTUs retrieved in our primary species hypothesis (PSH) and 2) checking for putative additional species by calculating the speciation probabilities for each population (separating putative sympatric cryptic species uncovered in the phylogenetic approach into separate populations). To evaluate the effect of user-incorporated prior values we tested four different prior combinations allowing for large vs. small ancestral population sizes and deep vs. shallow divergence times (see Methods for details). When using the twelve MOTUs from our PSH as a guide tree, most nodes were supported by posterior probabilities (PP) of 1.0 (i.e., 100% of the applied speciation models supported the two lineages of the specific node as species) (Figure 3A). We consider a speciation probability value of ≥ 0.95 as strong support for a speciation event, which is recovered for all identified MOTUs except for the speciation event between MOTU X and XI (PP 0.90-0.96, Figure 3A). The latter event however received consistent support ≥ 0.95 in the second set of analyses in which each population was treated separately (Figure 3B). BPP also indicated high support for a split within MOTU XI (*P. milaschewitchii*) between populations from the Mediterranean and Black Sea; however these results were ambiguous among analyses. In general, assumed small ancestral population size and long divergence times resulted in the highest speciation support values (Figure 3 in green), while large ancestral population sizes and long divergence times resulted in the lowest PPs (Figure 3 in blue). Shallow divergence times also provided better support values (Figure 3 in red) but are considered unlikely based on molecular clock data of *Pontohedyle*[53]. In summary, BPP resulted in 13 MOTUs with PP ≥ 0.95 (see Figure 2J).

**Figure 4:**
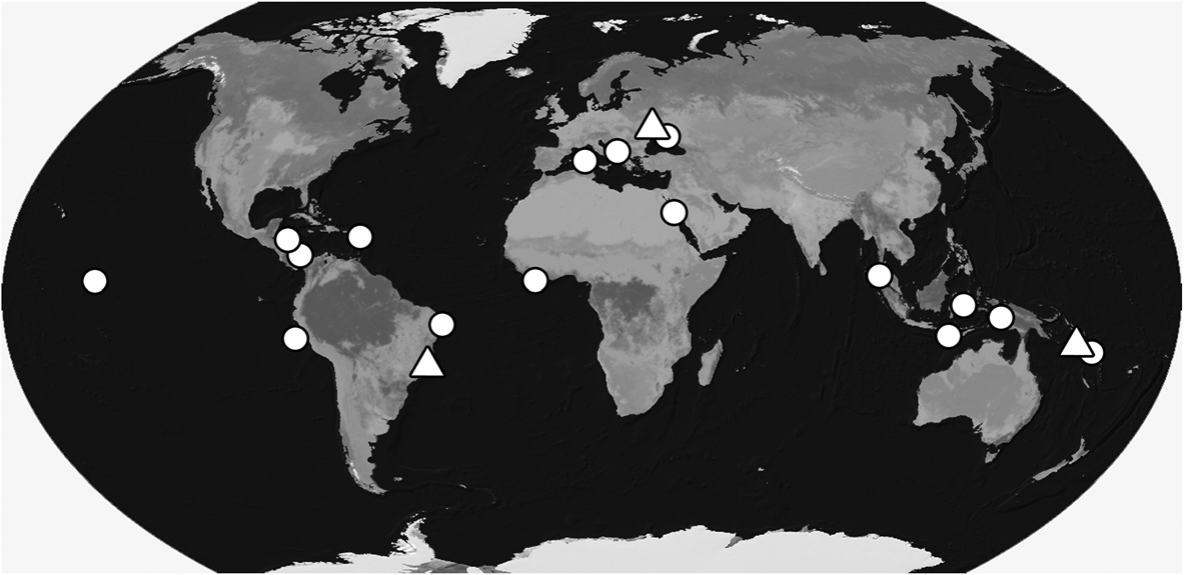
**Map of sampling sites for *Pontohedyle*.** Type localities of described *Pontohedyle* species (white triangle) and own collecting sites (white dots). For details on localities and habitat description see Table 1).

#### Pairwise distances and Automatic Barcoding Gap Discovery (ABGD)

Applying the ABGD with the standard settings resulted in one partition (i.e., no barcoding gap) in both our COI and 16S dataset. When lowering the X-value (relative width of barcoding gap) to 1.2, ABGD clustered the sequences into 10 MOTUs for COI (incomplete dataset, see Figure 2K) with a prior of intraspecific divergence up to 0.0359, and 11 MOTUs for 16S rRNA (Figure 2L) with a prior of up to 0.0599, which was congruent with the PSH. The 16S rRNA results, however, contradicted all other approaches applied here in not splitting the ambiguous MOTU VIII into two separate entities (which was strongly supported in BPP analyses). ABGD results were independent from the chosen model (Jukes-Cantor and Kimura) and unaffected by changes of prior limit of intraspecific variation.

Species Identifier was then used to calculate the intra- and interspecific variability within the MOTUs. Choosing our PSH as *a priori* species input for pairwise distance calculation, clusters were in agreement at a threshold from 14.7% - 18.5% for COI and 11.2% - 18.9% for 16S rRNA. Repeating the analyses and subdividing the ambiguous MOTU VIII into two putative candidate species clusters were in full agreement starting at a threshold of 8.8% for COI and 6.3% for 16S rRNA. Within the MOTUs, the largest intraspecific uncorrected p-distances occurred within the ambiguous MOTU VIII with 14.65% for COI (n = 3) and 14.47% for 16S rRNA (n = 4), followed by ‘*P. brasilensis*’ (MOTU XII) with 8.7% for COI (n = 4), and *P. verrucosa* (MOTU VI) with 7.1% for 16S rRNA (n = 4). Among the other clades, the largest uncorrected intraspecific p-distances were lower, ranging from 1.83 - 5.03% for COI and 0.22 - 4.45% for 16S rRNA. Between MOTUs, the smallest interspecific p-distances were larger than the intraspecific variation; i.e., 18.32% for COI (between Western Atlantic MOTU II and Indo-Western Pacific *P. verrucosa* (MOTU VI)) and 14.69% for 16S rRNA (between Red Sea MOTU IV and Indian Ocean MOTU V), whereas the smallest mean interspecific p-distances within our dataset were 24.68% for COI and 28.58% for 16S rRNA. With a fixed threshold of 11% – recorded as mean sequence divergence for COI in congeneric species pairs in Mollusca [54] – applied to our (incomplete) COI dataset, Species Identifier recovered 10 clusters in comparison to the other species delineation approaches (Figure 2M).

#### Secondary species hypothesis (SSH)

Our SSH is based on a minimum consensus approach (see Figure 2N, Material and Methods and detailed discussion below) across species delineation approaches. It was identical to our PSH and suggested at least 12 mainly cryptic candidate species, three of which correspond to existing names in nomenclature. *Pontohedyle* sp. 6 (corresponding to MOTU VIII), however, remains problematic, since nearly all molecular species delineation approaches split this unit into a minimum of two independent lineages (with high support, see e.g., Figure 3B); only the ABGD analysis based on 16S rRNA did not support this split.

## Discussion

### Molecular species delineation in elusive taxa

Our study demonstrates that traditional taxonomic treatment is not efficient for uncovering the true diversity in meiofaunal *Pontohedyle* slugs. It is essential to have an operational molecular-based concept for species delineation (DNA taxonomy) and species re-identification that informs future ecological, biogeographical or conservation approaches. The methods should be cost-efficient and fast, but in the first place they need to be reliable, and able to deal with rare (or rarely sampled) meiofaunal species elusive to population genetics.

Puillandre et al. [55] proposed a workflow for large-scale species delineation in hyperdiverse groups, starting with a COI barcoding dataset analyzed with ABGD and GMYC which leads to the primary species hypothesis (PSH). Independent information (from other molecular markers, morphology and ecological traits) is subsequently added to lead to the secondary species hypothesis (SSH) [55]. This formalized strategy [55] is linear, starting with pre-selecting samples according to a PSH that depends on a single mitochondrial marker, before further information is added that might expand or contradict the PSH. What is an efficient workflow for large-datasets with dense sampling coverage and thus high-quality COI barcoding output, may be inapplicable for datasets in little known and putatively under-sampled taxa. The latter would benefit from full consideration of all information already available for a PSH, and a parallel, combined approach of multiple markers and multiple delimitation methods. Especially when it is unfeasible to sample multiple specimens, multiple loci lead to more reliable results [22]. Multi-marker barcoding provides an *a posteriori* double-check for contamination, sequencing errors or mitochondria-specific pitfalls (e.g., the presence of numts or mitochondrial introgression), and the idiosyncrasies of individual markers [16,56]. Our study shows that COI analyses perform well on our dataset but due to amplification problems applying universal COI barcoding primers, three candidate species would have remained unconsidered. Multi-marker barcoding makes better use of rare specimens.

Our global sampling is sparse rather than comprehensive, including a few singletons from distant populations. Nevertheless, we are able to propose a primary species hypothesis on the evidence of a molecular phylogeny and concordance in reciprocally monophyletic entities (Figure 1A). We use concordance to mean two things: 1) in agreement among different phylogenetic analyses (ML, parsimony, Bayes), to account for the risk in false species delimitation due to errors in phylogenetic reconstruction [22,38]; and 2) in agreement among single-gene trees (two mitochondrial markers, one nuclear) and the concatenated dataset, to avoid false signals due to recent population genetic processes [16,22].

In this phylogenetic approach as starting point for further analyses, we consider relative branch lengths as proxies for evolutionary distance. Reciprocal monophyly as a criterion for species delineation is being criticized as too stringent [22], since monophyly of species in gene trees is not assured if lineages are not fully sorted [16,57,58]. Although the absence of reciprocal monophyly might not be sufficient for lumping species, its presence with several independent markers indicates compatibility of gene and species trees and can thus be used for a PSH, especially when combined with approaches capable of detecting recent speciation processes (like BPP [22,38]). As an example for molecular species delineation in poorly known taxa, our study benefits from divergence times, which in *Pontohedyle* is estimated to have started in a late Mesozoic timeframe ([53], KMJ own unpublished data). Thus, recent radiations, which present a major pitfall for molecular delineation approaches [12,16,34,57], are unlikely to hamper overall delineation success in our study; exceptions and problematic cases are discussed below. Plotting available data from morphology, biogeography and hydrographic features at least partially allows the diagnosis of the MOTUs beyond their molecular characteristics.

To date, the analyses of pairwise distances, with application of generalized universal thresholds (e.g., [9,54]) or relative thresholds (e.g., 10× rules, requiring the interspecific variability to be at least ten times the variability of intraspecific variation [59]) is the most commonly used form of molecular species delineation. This approach has however been widely criticized as arbitrary due to high variation and frequent overlap of intra- and interspecific variation [12,14,16,17,34,60]. This criticism also affects the concept of a ‘barcoding gap’, i.e., a significant gap in the distribution of intra- vs. interspecific variation [12]. Intrapopulation variation might, in fact, exceed divergence between species [61], which has been demonstrated in well-sampled groups with reliable independent datasets for species delineation such as karyology [13]. It is evident that intra- and interspecific variation are biased by sampling coverage [17] and there is a high risk of misidentification, especially in undersampled phylogenies [12]. Applying a fixed threshold of 11%, which has been determined as the mean sequence differentiation between species pairs in Mollusca [54], yields the same clusters as our complex delineation approach. However, we consider the good performance of a fixed threshold as random and due to the fact that this applies a mean distance. Using the smallest interspecific distances (as recommended by Meier et al.[62]) might- logically smaller than 11% -lead to an overestimation of species richness in our dataset. Moreover, these pairwise distance approaches do not serve as an independent tool for species delineation, but depend highly on pre-defined species limits. Using our PSH as the *a priori* species hypothesis we detect a barcoding gap, which, however, shifts considerably when the e.g., MOTU VIII is additionally split into two entities, demonstrating the circularity of the approach. The ABGD method [39] still suffers from limitations based on the genetic distance concept and barcoding gap discussed above, but presents a major advantage since it is applicable as an independent tool without an *a priori* species hypothesis. ABGD analyses may be problematic on small datasets with less than three sequences per species [39]. When the standard settings of ABGD were applied to our dataset, it failed to partition our dataset based on pairwise distances. Lowering the user-defined relative-gaps width (X) enabled ABGD to recover clusters that are congruent with the other delineation approaches for both mitochondrial markers. Although we present the first study on meiofaunal slugs with representative worldwide taxon sampling, we know our dataset is likely to represent only a fragment of the hidden diversity in the genus because a) tropical sands still are largely unsampled, b) suitable habitats are patchy and disjunct, and c) the indication of accumulated diversity in geographically small areas (e.g., three distinct genetic *Pontohedyle* lineages on the island of Moorea). Thus, the discovered ‘barcoding gap’ may be an artifact of limited sampling.

A series of independent tools of molecular species delineation have been developed recently [21,22,32,33,38], but only few studies have tested these comparatively. In a thorough comparison, Sauer & Hausdorf [16] report that Gaussian Clustering [21] yielded the best performance in relation to morphological species delineation, but several sequences per population are needed to recognize reliably a separate cluster (i.e., candidate species) [16]. In contrast, the GMYC-model has shown, in a series of studies, ability to discriminate effectively between coalescent and speciation patterns. These matched species identified via independent criteria (e.g., independent molecular markers, morphology, geography) [32,33,35,55,63], even in groups with little sampling coverage [36]. In our study GMYC congruently recognizes the same MOTUs as separate entities as our PSH and thus does not tend to oversplit data, as suspected previously [18]. Sauer & Hausdorf [16] demonstrated considerable differences in GMYC results depending on the method of reconstruction of the ultrametric input tree. While this stresses the need to test the effect of different input trees, we consider the risk in our dataset as minor because all our phylogenetic approaches recover similar topologies. We conclude that GMYC in general is a delineation tool capable of dealing with data from poorly-sampled groups.

Even though the performance of BPP dramatically improves when sampling at least five sequences per population, it achieves correct assignment of models also in small datasets (simulated, empirical and including singletons) when a high number of loci is used [38]. It is especially useful to detect cryptic species in sympatry [22,38,64]. Our BPP analyses supported all MOTUs identified in our PSH and, additionally, split the ambiguous MOTU VIII into two entities, in agreement with GMYC analyses. Given the possibility of testing the speciation support for each of the sampled populations by incorporating prior information on population size and divergence times, BPP is especially useful to avoid lumping of species. However, in its present form it is limited to dealing with small datasets (max. 20 species).

In the present study statistical parsimony congruently recovered the same MOTUs of the PSH, but considerably oversplit the data in comparison to the other methods (see Figure 2H-I). Whereas GMYC also resulted in additional splits and some populations in BPP resulted in ambiguous PPs (see Figure 2B-G, Figure 3B) that are potentially related to recent speciation processes, the comparative oversplitting in TCS might rather be an artifact of high substitution rates on mitochondrial markers [16], as reported from several other molluscan taxa (e.g., [14]).

Although all MOTUs based on singletons (but with the complete dataset of all three markers available) are clearly separated into independent lineages in all different approaches (see Figure 2), inconsistencies arose within the dataset for MOTUs containing 5–10 specimens (see e.g., MOTU VI, XI, XII in Figure 2), which clustered into one or more entities in different analyses. However, speciation is a continuous process [65,66] and delineation approaches offer only a snapshot of this process, so we expect to encounter various stages of differentiation. Recent radiations leading to incomplete lineage sorting might explain ambiguous results on different mitochondrial markers (see e.g., Figure 2H-I MOTU VI and XII) and more data and population genetic approaches are needed to reveal the genetic structure within these entities.

Most of the molecular species delineation methods currently available are not designed to incorporate the common phenomenon of rarity (i.e., species only represented by singletons or few sequences) [28]. Sampling efforts by us and colleagues confirm that *Pontohedyle* and many other meiofauna taxa truly are rare and can be expected to have small effective population sizes. Thus, we consider a integrative approach as most suitable for molecular species delineation in little known, putatively widespread and notoriously under-sampled taxa such as meiofauna in remote areas. Available methods of species delineation should be applied in parallel on different suitable barcoding markers (mitochondrial and nuclear markers) and combined with phylogenetic analyses that allow mapping of additional information from morphological and ecological traits. We chose a minimum consensus approach across all methods, conservatively relying only on fully corroborated entities. Sauer & Hausdorf [16,38] noted an oversplitting in all different species delineation approaches when these are compared to morphological analyses that include characters directly involved in speciation patters (i.e., morphology of genitalia). We aim to minimize the risk of oversplitting (i.e., inclusion of false positives), and rather put up with the risk of false negatives (i.e., lumping multiple species into one) and not detecting, yet, the entire diversity present in our dataset. Moritz [67] argued that false positives, because they are highly divergent genetically, might still present important components of biodiversity. We agree but their inclusion causes an incalculable taxonomic inflation and might lead to misinterpretations of meiofaunal biogeography and evolution. In our study, the minimum consensus approach is feasible, since results are not contradictory in recovering different entities (Figure 2N), probably due to long periods of reproductive isolation. Our scheme, however, is not applicable to studies with ambiguous results, which would call for further lines of evidence and thorough evaluation of the contradictions before delineation of candidate species could be achieved.

### Species delineation in *Pontohedyle*

Our results revealed a secondary species hypothesis of twelve distinct species, diagnosable by multiple methods. Morphological characters traditionally used for species delineation in Acochlidia, split the worldwide sampled *Pontohedyle* populations into only two morphospecies: *P. milaschewitchii* (lateral radula tooth with denticle, Figure 1E) and *P. verrucosa* (lateral radula tooth smooth, Figure 1D). Previously used external morphological characters such as the shape of oral tentacles, body length and width, or color of the digestive gland (e.g., [68]) depend highly on the stage of contraction and nutrition, and are variable through time for each individual [40,41] and therefore inappropriate for species delineation. *Pontohedyle* typically bear monaxone, rodlet-like spicules distributed randomly and frequently accumulated between the oral tentacles [40,69,70], which is confirmed here for members of both major clades of *Pontohedyle*. Although the presence of certain types of spicules is diagnostic for acochlidian genera or species, their absence (e.g., as in MOTU VII) is not, because it can be caused by environmental influences [71]. Using the presence of externally clearly visible eyes as a delineating character would lead to the identification of two more morphospecies (Figure 1A): one with smooth lateral radula tooth and externally clearly visible eyes (corresponding to MOTU I, distinguished from *P. verrucosa,* which lacks visible eyes) and one without externally visible eyes and with a denticle on the lateral radula tooth (in our phylogeny clustering among *P. brasilensis* with visible eyes). However, the presence of externally visible eyes depends on the degree of pigmentation, and was shown to be highly variable intraspecifically in other acochlids [72]. This is confirmed by our phylogenetic and molecular analyses, which clustered both ‘eyeless’ and eye-bearing individuals in ‘*P. brasilensis’* and *P. verrucosa* (see Figure 1A).

In the light of our phylogenetic hypothesis, a convergent modification of the lateral radula tooth has taken place within the *P. verrucosa* clade in the two intertidal MOTUs I and VI. The power of morphological species delineation is the potential to include characters directly involved in the speciation process, e.g., from the reproductive system [16]. Based on previous histological comparisons, Jörger *et al.*[41] failed to find any morphological characters justifying discrimination between the closely related western Atlantic ‘*P. brasilensis*’ (MOTU XII) and its Mediterranean congener *P. milaschewitchii* (MOTU XI). Details on the reproductive system of *P. verrucosa* are missing in the original description [73], but own histological comparisons using 3D reconstruction based on serial semi-thin sections from material collected at the type locality revealed no major differences (KMJ, own unpublished data). Thus, even sophisticated micro-anatomical comparisons seem unpromising for species delineation in these highly simplified and uniform slugs.

In general, morphology in meiofaunal organisms is characterized by extensive parallelism and convergent adaptations to the mesopsammic environment [44,74], which frequently results in low interspecific morphological variability [7]. This is true of the microhedylacean Acochlidia, which are exclusively found in interstitial spaces in sediment, and show a tendency toward reduction of complexity in major organ systems [7]. In contrast, hedylopsacean Acochlidia, whose evolution involves several habitat shifts from marine interstitial to amphibious or freshwater benthic habitats, subsequently possess complex excretory and reproductive systems (e.g., [75-78]). Generally, there is little morphological variation in all major organ systems even at family- and genus-level see [7], but the morphological uniformity in global *Pontohedyle* is most striking. With its vermiform body, a putatively multi-functional radula, ‘simplified’ organ systems and a special fast and imprecise mode of sperm transfer [79], *Pontohedyle* reflects a meiofaunal slug lineage highly adapted to its interstitial habitat (see discussion below).

Integrating available data on morphology and ecology to the most conservative of our molecular results, the minimum consensus approach (see Figure 2N), suggests that *Pontohedyle* represents a newly-discovered radiation of cryptic species. This radiation consists of at least nine candidate species plus the confirmed valid species *P. milaschewitchii* (Kowalevsky, 1901) and *P. verrucosa* (Challis, 1970), plus *P. brasilensis* (Rankin, 1979), which is here reestablished as a valid taxon. In accord with recent findings for other microscopic taxa (e.g., [29,35,47,80]), our data indicates that expanded meiofaunal sampling in the future will likely uncover even more cryptic lineages. We agree with earlier authors [52] in the practical benefit and importance (e.g., for biodiversity assessments, and conservational and ecological concerns) of describing cryptic species to give them formal taxonomic validity, rather than retaining them as numbered candidate species. A formal description based on diagnostic molecular characters (DNA taxonomy in a strict sense) of all herein discovered candidate species is, however, beyond the scope of the present paper and will be documented in a subsequent taxonomic publication.

### Does the unveiling of cryptic species solve the meiofauna paradox?

For centuries taxonomy has depended on morphological distinctiveness. In the absence of distinguishing morphological characters many taxa (particularly meiofauna) were described as amphi-Atlantic or even cosmopolitan see (e.g., [51,52]). Due to the predicted low dispersal abilities and limited reproductive output, long-range distribution in meiofauna is known as the ‘meiofauna paradox’ [42]. In fact, recent re-examination has uncovered a series of radiations of cryptic species across different meiofaunal taxa (see e.g., [35,46-51,72]). Our molecular analyses show considerable geographic structure within global *Pontohedyle* and demonstrate that the putatively amphi-Atlantic meiofaunal slug, *P. milaschewitchii* (from the Mediterranean and including its Western Atlantic synonym *P. brasilensis*) represent cryptic sibling species, including the Eastern Pacific *Pontohedyle* sp. 9 (see Figure 1A, as MOTU X). Meanwhile, our data also confirms surprisingly wide ranges in distribution: in *P. brasilensis* (MOTU XII), with a range from southern Brazil to Belize (over 6500 km linear distance), or in *P. verrucosa* (MOTU VI) from the Pacific Solomon Islands to Indo-Pacific Indonesia (approx. 5000 km linear distance). The same scenario of long-distance dispersal on the one hand and clear spatial structuring on the other have also been recorded in other meiofaunal taxa; e.g., Nematoda [81], Nemertea [29,82] and Rotifera [45]. The widespread MOTUs in the present study span predicted barriers of gene flow for minute meiofaunal taxa, such as the Amazon freshwater and sediment plume or deep-sea regions between islands. With a typically low reproductive output in Acochlidia (max. of 40 eggs in *P. milaschewitchii*, KMJ pers. obs.), free veliger larvae are assumed to stay in the interstices of the sand grains rather than entering the water column [74] thereby avoiding long distance dispersal. Fertilized eggs are attached to sand grains (KMJ, pers. obs.) and might promote dispersal via current driven sediment transport along shorelines [42]. Data from other meiofaunal taxa suggest that the adult rather than larva serve as the main dispersal stage [83-85]. Dispersal by actively entering the water column as observed, e.g., in copepods [85] is considered improbable in soft-bodied acochlidian slugs [71], but accidental suspension (e.g., caused by waves, currents or tropical storms) and transport in the water column could allow a step-wise distribution along continuous coastlines and thus explain large range distribution [83] as observed in *P. brasilensis*. Neither our phylogenetic analyses (Figure 1A) nor Bayesian Species Delineation (Figure 3B) offered evidence that geographical barriers (as e.g., the Amazonas basin) constitute a distribution barrier for these meiofaunal slugs, as the Brazilian *P. brasilensis* clustered among individuals from the Caribbean (BPP only split Black Sea and Mediterranean populations of *P. milaschewitchii* into two entities, however with ambiguous support between analyses). Comparably high genetic distances from mitochondrial markers between the geographically separated populations especially in *P. verrucosa* and *P. brasilensis* and unconnected haplotype networks (Figure 2H-I) might indicate recent reproductive isolation and genetic diversification. More sensitive molecular markers (e.g., AFLPs) and more material are needed for thorough population genetic approaches to reveal the genetic structure in widespread meiofaunal slugs.

In the absence of a fossil record for meiofaunal slugs the only available estimate for divergence times derives from a molecular clock approach calibrated with shelled heterobranch fossils. Jörger *et al.*[53] estimated the origin of the genus *Pontohedyle* to the late Cretaceous, 84 mya (95% confidence interval ranging from 160–60 mya), providing a rough estimation of how much time was available for diversification and circum-global dispersal of *Pontohedyle* slugs.

### Origin and diversification of *Pontohedyle*

The genus *Pontohedyle* shows a circum-tropical distribution with a single derived species (Mediterranean/ Black Sea *P. milaschewitchii*) inhabiting temperate waters (see Figure 1A), confirming general trends of highest species diversity in tropical sediments [1]. Although the distribution of co-existing Mediterranean acochlids like *Microhedyle glandulifera* or *Hedylopsis spiculifera* extends northwards on the European Atlantic Coast, recorded up to 59° latitude ([71], own unpublished data), *Pontohedyle* has never been found in colder waters despite a well-studied meiofauna and hydrographic conditions similar to the Mediterranean. The distribution of *Pontohedyle* might be constrained by ancestry from warm-water adapted animals.

Considering the estimated mid to late Mesozoic origin [53] and the recent primarily tropical distribution pattern in *Pontohedyle,* it is most likely that this meiofaunal slug clade originated in Tethyan waters. The tropical radiation in both *Pontohedyle* clades (see Figure 1A) reveals a mixture of Western Atlantic and Indo-Western Pacific entities with single Eastern Atlantic or Eastern Pacific lineages. Such a complex distributional pattern points to a complex historic biogeographic scenario: large-scale geological events, such as the separation of the Atlantic Ocean and the Indo-West Pacific province, sealed in the closure of the Tethys seaway in the early Miocene [86] followed by a series of vicariant events (of tectonic and climatic origin) during the Cenozoic that affected the global tropical ocean [87]. All of these likely contributed to allopatric speciation in *Pontohedyle*. Due to the predicted low dispersal abilities in meiofaunal taxa, relatively small-scale geological disruptive events (via landslides or formation of rivers) might form a (temporary) barrier for gene flow, proving time for ecological diversification and reproductive boundaries to evolve. Two species (*Pontohedyle* sp. 2 and *P. brasilensis*) were collected at the same localities (WA-1, WA-4, WA-7, see Table 1). Sympatric speciation might be common in the marine environment [88] and especially in the mesopsammic habitat, which is highly structured by gradients in chemistry, type and quantity of food resources or predation, thereby forming numerous ecological micro-niches within small areas (see e.g., [89]). Differences in the histology of the digestive gland content (KMJ, pers. obs.), potentially correlated with the lack of denticle on the lateral radula tooth, indicate putative different food sources and ecological micro-niches in *Pontohedyle* (e.g. in *P. verrucosa* and *Pontohedyle* sp. 1).

**Table 1:** **Details on sampling localities and habitat description for *Pontohedyle *analysed in the present study**

**Field code**	**Locality**	**Water body**	**GPS**	**Depth**	**Habitat remarks**
BS-1*	Sebastopol, Ukraine, Europe	Black Sea	-	8 m	subtidal, coarse sand
MS-1	Cape Kamenjak, Istria, Croatia, Europe	Mediterranean Sea	N 44°46’04” E 13°54’58”	6-9 m	subtidal, between rocks, exposed, coarse sand
MS-2	Rovinj, Istria, Croatia, Europe	Mediterranean Sea	N 45°07’05” E 13°36’58”	3-4 m	subtidal, sand patches between rocks and sea grass, coarse sand
MS-3	Calvi, Corse, France, Europe	Mediterranean Sea	N 42°33’57” E 08°44’15”	22 m	subtidal, sand patches between seagras, coarse sand/ shell grid
EA-1	MiaMia, Ghana, Africa	Gulf of Guinea, East Atlantic Ocean	N 04°47’46” W 02°10’06”	2-3 m	subtidal, fine sand
WA-1	near Castries, St. Lucia, Central America	Caribbean Sea, West Atlantic Ocean	N 14°3’34.56” W 60°58’18.24”	2-3 m	subtidal, sand patches between seagras, coarse sand
WA-2	Soufriere Bay, St. Lucia, Central America	Caribbean Sea, West Atlantic Ocean	N 13°51’24” W 61°03’58”	8-9 m	subtidal, sand patches between coral blocks, coarse sand
WA-3	Carrie Bow Cay, Belize, Central America	Caribbean Sea, West Atlantic Ocean	N 16°48’13.44” W 88°4’36.9”	31 m	subtidal, exposed sand plain, relatively fine sand
WA-4	Carrie Bow Cay, Belize, Central America	Caribbean Sea, West Atlantic Ocean	N 16°48‘13.44“ W 88°4‘36.9“	15 m	subtidal, sand patches between corals, coarse sand
WA-5	Carrie Bow Cay, Belize, Central America	Caribbean Sea, West Atlantic Ocean	N 16°48‘ 8.94“ W 88°4‘47.1“	3-5 m	subtidal, exposed, coarse sand
WA-6	Curlew Reef, Belize, Central America	Caribbean Sea, West Atlantic Ocean	N 16°47‘24.96“ W 88°4‘43.38“	2 m	subtidal, sand patches between corals exposed to waves, coarse sand
WA-7	Carrie Bow Cay, Belize, Central America	Caribbean Sea, West Atlantic Ocean	N 16°48‘7.62“ W 88°4‘36.42“	14-15 m	subtidal, sand patches on ridge, coarse sand
WA-8	Carrie Bow Cay, Belize, Central America	Caribbean Sea, West Atlantic Ocean	N 16°48‘7.62“ W 88°4‘36.42“	31 m	subtidal, protected trough inside ridge, coarse sand
WA-9	Bocas del Toro, Panama, Central America	Caribbean Sea, West Atlantic Ocean	N 9° 21' 2.34" W 82° 10' 20.7"	5-8 m	subtidal, protected, coarse sand
WA-10	off Recife, Brazil, South America	South West Atlantic Ocean	S 8° 3' 17.34" W34° 47' 40.38"	20 m	subtidal, relatively fine coral sand
RS-1	Sha’abMalahi, Egypt, Africa	Red Sea	^++^) N 24°11‘50“ E 35°38‘26“	20 m	subtidal, relatively fine coral sand
IO-1	KoRacchaYai, Phuket, Thailand, Asia	Andaman Sea, Indian Ocean	N 7°36‘15“ E 98°22‘37“	6-7 m	subtidal, relatively fine coral sand
IO-2	KoRacchaYai, Phuket, Thailand, Asia	Andaman Sea, Indian Ocean	N 7°36‘15“ E 98°22‘37“	20-22 m	subtidal, coarse sand, exposed
IP-1	Pulau Moyo, Nusa Tengarra, Indonesia	Flores Sea, Indian/ PacificOcean	S 8°13‘59“ E 117°28‘32“	5-6 m	subtidal, coarse coral sand
IP-2	Pulau Banta, Nusa Tengarra, Indonesia	Flores Sea, Indian/ PacificOcean	S 8°23‘58“ E 119°18‘56“	5-6 m	subtidal, coarse coral sand
IP-3	Pulau Banta, Nusa Tengarra, Indonesia	Flores Sea, Indian/ PacificOcean	S 8°23‘58“ E 119°19‘01“	0-1 m	intertidal, coarse coral sand
WP-1	Lembeh Strait, Sulawesi, Indonesia	Banda Sea, West Pacific Ocean	N 1°27‘53“ E 125°13‘48“	3-5 m	subtidal, between coral blocks, exposed, coarse sand
WP-2	Misool, Raja Ampat, Indonesia, Asia	Ceram Sea, West Pacific Ocean	S 2°14’53.46” E 130°33’18.42”	0-1 m	intertidal, protected beach, coarse, coral sand
WP-3*	Komimbo Bay, Guadalcanal, Solomon Islands, Oceania	West Pacific Ocean	S 9°15’50.58” E 159°40’5.82”	0-1 m	intertidal, protected beach, coarse, coral sand
WP-4	Honiara, Guadalcanal, Solomon Islands, Oceania	West Pacific Ocean	S 9°25'43.29'' E 159°56'57.24''	0-1 m	intertidal, protected beach, coarse, coral sand
CP-1	E of Cook’s Bay Pass, Moorea, Oceania	Central Pacific Ocean	S 17°28’33.96” W 149°49’51.6”	10-11m	subtidal, coarse sand, shell grit and rubble
CP-2	E of Cook’s Bay Pass, Moorea, Oceania	Central Pacific Ocean	S 17°28’17” W149°48’42”	18-20 m	subtidal, coarse sand, shell grit and rubble
CP-3	Motu Iti, Moorea, Oceania	Central Pacific Ocean	S 17°32’50.172” W 149°46’35.4”	3-4 m	subtidal, fine to medium coral sand
EP-1	Punta Sal, Peru, South America	East Pacific Ocean	S 3°58’55” W 80° 59’10”	8 m	subtidal, coarse sand

^++^) approximation from Google Earth.

* marks the localities, which correspond to the type localities of valid *Pontohedyle* species.

It remains stunning that circum-tropical dispersal and speciation processes in *Pontohedyle* over a long evolutionary timeframe (i.e., Mesozoic [53]) are not reflected in morphological differentiation. This extreme case of morphological stasis and similar reports from other meiobenthic groups (e.g. [90]) might be explained in the light of the main physical constraints of the interstitial environment: This habitat is unstable at very short timescales (e.g., due to wave action, currents or storms) and can be split into numerous micro-niches, allowing for changes in ecological, physiological and behavioral traits. However, these are not necessarily reflected in morphological changes and the mesopsammon might be highly stable in an evolutionary perspective. Our results on *Pontohedyle* slugs show that a well-adapted body plan can be conserved for millions of years in a worldwide evolutionary success story.

## Conclusions

Combining traditional taxonomic, hydrographic and geographic evidence with multi-marker phylogenetic and multiple species delineation approaches herein allowed us to refute a cosmopolitan and amphi-oceanic distribution of *Pontohedyle* species. Uncovering a radiation of cryptic species partially solves the meiofaunal paradox. Remaining long-range distributions in some *Pontohedyle* species might indicate that the dispersal abilities of meiofaunal slugs are currently underestimated, or that our approach is unsuitable of detecting an even higher degree of cryptic radiation in recent times. Overall, *Pontohedyle* presents a stunning example of extreme morphological stasis and uniformity over long evolutionary timeframes, probably constrained by their simplified bodyplan and by the requirements of the meiofaunal habitat.

Our study boosts diversity in *Pontohedyle* sea slugs from 3 nominal to 12 (candidate) species, and confirms the taxonomic deficit in the mesopsammic fauna. It suggests an unexpected magnitude of diversification and cryptic speciation still exists in other small-sized, neglected taxa. Our workflow of delineating minute and highly cryptic *Pontohedyle* species included integrating phylogenetic, traditional taxonomic and any other relevant evidence towards a primary species hypothesis. This was then evaluated and refined by the consensus evidence from a selection of molecular species delineation methods, including ABGD, statistical parsimony, GMYC and Bayesian species delineation. Both latter methods can deal with rarity as is also confirmed herein. In the age of the biodiversity crisis, we need an efficient and reliable way of addressing species diversity in rare and elusive species. Our workflow still only provides a conservative estimation on species diversity and tolerates the risk of false negatives; we still hope it can serve as a starting point to uncover the hidden diversity of elusive taxa, regardless whether coastal, mesopsammic, deep sea or terrestrial.

## Methods

### Sampling and fixation

The sampling effort for *Pontohedyle* was conducted worldwide, resulting in specimens from 28 collecting sites in temperate and tropical zones. Samples include re-collection from the type localities of valid *Pontohedyle* species for taxonomy see [7,91]: *P. milaschewitchii*[69] and *P. verrucosa*[73]. ‘*P. brasilensis*’ was considered a junior synonym of *P. milaschewitchii* based on morphological data [41] and was recollected near the original locality (see Figure 4). For detailed data on localities, depth and habitat descriptions see Table 1. Subtidal sands were collected via snorkeling or SCUBA diving. *Pontohedyle* were extracted from intertidal and subtidal sand samples following the method described by Schrödl [92] using a MgCl_2_/ seawater solution for anesthetization. For molecular work, specimens were fixed in 96–99% ethanol. Voucher specimens were preserved in FSW or 4% glutaraldehyde after relaxation in MgCl_2_ solution to prevent retraction.

### Morphological comparison

All specimens were documented alive under a dissecting microscope and whenever possible analyzed under a light-microscope for spicules and radula characteristics prior to fixation. Radulae of groups defined by molecular data were analyzed by light- and scanning electron microscopy (SEM). Radulae from specimens from EP-1, CP-2 and CP-3 could unfortunately not be recovered from DNA extraction and were unavailable for further study. To separate the radulae from the surrounding tissue, entire specimens were dissolved in a solution of 45 μl ATL (tissue lysis) buffer and 5 μl Proteinase K (derived from the Qiagen DNeasy Blood and Tissue Kit) overnight at 56°C. Radulae were rinsed in Millipore-purified water, studied with a Leica DMB-RBE microscope (Leica Microsystems, Germany) and photographed with a SPOT CCD camera (Spot Insight, Diagnostic Instruments, Inc., USA). Following light-microscopical examination, radulae were transferred onto SEM stubs with self-adhesive carbon stickers and coated in gold with a Polaron Sputter Coater E5100 for 120 sec. SEM examination was carried out using a LEO 1430VP SEM at 15 kV.

### DNA extraction, amplification and sequencing

Genomic DNA was extracted from entire specimens using the DNeasy Blood and Tissue Kit (Qiagen) or the NucleoSpin Tissue Kit (Macherey-Nagel GmbH & Co), following the manufacturer’s instructions. DNA vouchers are stored at the DNA bank of the Bavarian State Collection for Zoology (ZSM; http://www.dnabank-network.org, see Additional file 2 for accession numbers). Three markers were amplified using polymerase chain reaction (PCR): partial nuclear 28S rRNA (approx. 950 bp) and partial mitochondrial 16S rRNA (approx. 440 bp) and Cytochrome *c* Oxidase subunit I (COI) (approx. 655 bp), using primers and PCR protocols listed in Jörger *et al.*[53]. Successful PCR products were purified using ExoSap IT (USB, Affimetrix Inc.; for 16S rRNA and COI) and the NucleoSpin Extract II (Macherey-Nagel GmbH & Co, for 28S rRNA). Cycle sequencing using Big Dye 3.1 and the PCR primers was conducted by the Genomic Service Unit of the Department of Biology, Ludwig-Maximilians-University Munich, as well as the sequencing reaction on an ABI 3730 capillary sequencer.

### Phylogenetic analyses

Consensus sequences from forward and reverse strands were created and edited using Geneious Pro 5.4.2 [93]. All sequences generated in this study were checked for potential contamination using BLAST searches [94] in GenBank (http://blast.ncbi.nlm.nih.gov/Blast.cgi). Alignments for each marker were generated with Muscle [95] using the default settings. To avoid misleading signal from hard to align regions, ambiguous parts of the 28S and 16S rRNA alignments were removed using Gblocks [96] with settings for a less stringent selection and analyzed comparatively. The removed parts of the alignments (94 bp out of 471 in the 16S rRNA alignment, 49 bp out of 1036 in the 28S rRNA alignment) were carefully checked manually for putative diagnostic signal such as insertions. The COI alignment was checked manually according to amino acids for stop codons and potential shifts in reading frame. Maximum likelihood single-gene trees of each marker (28S rRNA, 16S rRNA, COI) and multi-gene trees of the concatenated dataset were generated using RAxML v. 7.2.6 [97]. Models for nucleotide substitution were chosen with jModeltest [98], with five substitution schemes; i.e., choosing from 40 different models (GTR + G for 28S rRNA and COI and GTR + G + I for 16S rRNA). The RAxML analyses were conducted following the ‘hard and slow-way’ described in the RAxML 7.0.4 manual (using five parsimony starting trees, six different rate categories and generating 200 multiple inferences and 1000 bootstrap replicates). Additionally, we applied the ‘-d’-option (generating random starting trees) recommended for small datasets. The concatenated dataset was analyzed 1) without partitioning, 2) in two partitions (nuclear 28S rRNA and mitochondrial 16S rRNA + COI) and 3) in three partitions (corresponding to each marker) and topologies are compared. Outgroups (see Additional file 2) were selected based on the latest phylogenetic hypothesis for Acochlidia [7,53] and include members of all microhedylacean genera; *Asperspina brambelli* (Microhedylacea, Asperspinidae) was defined as the outgroup in phylogenetic analyses. For topological comparison we additionally generated a consensus tree with PAUP v 4.10 [99] applying maximum parsimony to the concatenated three marker dataset. All alignments and trees generated within this study are deposited to TreeBASE under project number 13633 (http://purl.org/phylo/treebase/phylows/study/TB2:S13633).

### Molecular based species delineation

We applied four different methods of molecular-based species delineation:

General Mixed Yule-Coalescent Model (GMYC) - a maximum likelihood approach as implemented in the ‘General Mixed Yule-Coalescent’ model (GMYC) was applied to discriminate between population and speciation processes and to identify species see [32,33]. Therefore, we generated ultrametric starting trees using BEAST 1.5.3 [100,101] from the COI and masked 16S rRNA alignments. Even though tested and designed for mitochondrial markers, for comparison we additionally calculated an ultrametric tree from the concatenated three-marker alignment (COI + 16S rRNA + 28S rRNA) which was also used for phylogenetic comparison. For the starting trees we performed relaxed lognormal clock analyses using the Yule prior and models for each marker specified above. We ran five independent Monte Carlo Markov Chains (MCMC) for 50 ×10^6^ generations each, sampling every 5000 steps. Single runs were combined with Log-Combiner 1.5.3 and checked for sufficient effective sampling size (ESS) in Tracer 1.5.3. Trees were combined using TreeAnnotator 1.5.3 with the first 10% of the trees discharged as burn-in. GMYC was performed in R using the SPLITS package (http://r-forge.r-project.org/projects/splits/) and analyses allowing single and multiple thresholds were performed.

Statistical parsimony - generating haplotype networks using statistical parsimony [37] is a common method derived from population genetics to visualize possible intraspecific relationships. Sequences are assigned to networks connected by changes, which are non-homoplastic with a certain probability. Even though this is not equivalent to defining species boundaries, statistical parsimony has also been applied successfully to delimit candidate species [16,33]. We generated haplotype networks with TCS 1.21 [102] applying a 95% parsimony criterion to both mitochondrial markers (COI and 16S rRNA) and nuclear 28S rRNA.

Bayesian species delineation – analysis was conducted using the program BP&P (BPP v2.1) [38,103] on the full three marker dataset. We ran two sets of BP&P analyses: 1) using our PSH as user-specified guide tree to evaluate the support of different speciation models for the identified MOTUs; 2) to test whether our PSH is too conservative and lumps species, we used a guide tree testing each population from different collecting sites as putative species. Putative sympatric cryptic species resulting in different MOTUs in our PSH were also separated into different populations. As prior information on ancestral population size (θ) and divergence times (τ) can affect posterior probabilities for speciation models [38,64], we tested 4 different prior combinations for each set: a) large ancestral population size, assigned gamma prior G(1,10) and deep divergences, root of the tree (τ) is assigned the gamma prior G(1,10), while the other divergence time parameters are assigned the Dirichlet prior ([38]: equation 2); b) small ancestral population size G(2,2000) and deep divergences G(1,10); c) large ancestral population size G(1,10) and shallow divergences G(2,2000); d) small ancestral population size G(2,2000) and shallow divergences G(2,2000). The latter cases are, however, considered evolutionary unlikely based on molecular clock estimates [53] of *Pontohedyle*. Since BP&P can currently only deal with up to 20 species, the population approach had to be conducted in several subsets. Each single analyses was run at least twice to confirm consistency between runs, run with two different algorithms and two different fine-tuning parameters. Since no biological data exists on ancestral population size in *Pontohedyle*, we consider it most objective to calculate the mean from different approaches and consider species with PP ≥ 0.95 as strongly supported by Bayesian species delineation.

Automatic Barcode Gap Discovery (ABGD) and pairwise distances – ABGD is an automated procedure that clusters sequences into candidate species based on pairwise distances by detecting differences between intra- and interspecific variation (i.e., barcoding gap) without *a priori* species hypothesis [39,55]. We used the web-server of ABGD http://wwwabi.snv.jussieu.fr/public/abgd/abgdweb.html and analyzed both mitochondrial markers using the two available models: Jukes-Cantor (JC69) and the Kimura K80 model. The program requires two user-specified values: P (prior limit to intraspecific diversity) and X (proxy for minimum gap width). To evaluate the effect on our dataset we tested X values from 0.1 to 5 and extended the maximum P value from 0.10 to 0.20.

Fixed thresholds – to calculate intra- and interspecific divergence among our detected MOTUs we used Species Identifier (obtained from Taxon DNA [17]) for both mitochondrial markers (COI and 16S rRNA), using the raw (unmasked) sequences. For comparison we tested the application of a fixed threshold of 11% for Mollusca suggested by Hebert et al. [54].

Minimum consensus approach - For our secondary species hypothesis (SSH, i.e., defining candidate species), we chose a conservative minimum consensus approach relying only on uncontradicted positive species identification based on the methods described above. Entities that were identified only by some of the approaches are thus ignored, giving equal priority to the applied methods.

## Competing interests

The authors declare that they do not have competing interests.

## Authors’ contributions

KMJ collected material, conducted molecular work and phylogenetic and species delineation analyses and drafted the manuscript. JLN organized sampling trips and supported molecular work. NGW dedicated valuable material to the study. All authors contributed to the discussion of the results and the final version of the manuscript. MS collected material, and planned and supervised the study. All authors read and approved the final version of the manuscript.

## Supplementary Material

Additional file 1 **Additional phylogenetic analyses of the concatenated and single-gene dataset (bootstrap values ≥ 50 given above nodes).** A. Maximum parsimony analyses conducted with PAUP on the concatenated three marker dataset. B. Maximum likelihood (ML) single-gene tree of nuclear 28S rRNA. C. ML single-gene tree of mitochondrial 16S rRNA (ambiguous parts in the alignment masked with GBlocks). D. ML single-gene tree of mitochondrial COI (due to extremely long branches *Asperspina brambelli* was considered as too distant and excluded from the analysis). Click here for file

Additional file 2 **Molecular data analyzed in the present study.** Museum numbers (ZSM – Bavarian State Collection of Zoology, SI – Smithsonian Institute (numbers refer to plate coordinates), AM – Australian Museum), DNA vouchers (all at ZSM) and GenBank accession numbers. Sequences retrieved from GenBank are marked with *. Click here for file

## References

[B1] Snelgrove PVR Getting to the bottom of marine biodiversity: sedimentary habitats - ocean bottoms are the most widespread habitat on earth and support high biodiversity and key ecosystem services Bioscience 1999 49 2 129 138 10.2307/1313538

[B2] Fonseca VG Carvalho GR Sung W Johnson HF Power DM Neill SP Packer M Blaxter ML Lambshead PJD Thomas WK Second-generation environmental sequencing unmasks marine metazoan biodiversity Nat Commun 2010 1 98 10.1038/ncomms1095 20981026 PMC2963828

[B3] Blaxter M Mann J Chapman T Thomas F Whitton C Floyd R Abebe E Defining operational taxonomic units using DNA barcode data Philos T R Soc B 2005 360 1462 1935 1943 10.1098/rstb.2005.1725 PMC1609233 16214751

[B4] Curini-Galletti M Artois T Delogu V De Smet WH Fontaneto D Jondelius U Leasi F Martínez A Meyer-Wachsmuth I Nilsson KS Patterns of diversity in soft-bodied meiofauna: dispersal ability and body size matter PLoS One 2012 7 3 e33801 10.1371/journal.pone.0033801 22457790 PMC3311549

[B5] Creer S Fonseca VG Porazinska DL Giblin-Davis RM Sung W Power DM Packer M Carvalho GR Blaxter ML Lambshead PJD Ultrasequencing of the meiofaunal biosphere: practice, pitfalls and promises Mol Ecol 2010 19 4 20 20331766 10.1111/j.1365-294X.2009.04473.x

[B6] Westheide W Progenesis as a principle in meiofauna evolution J Nat Hist 1987 21 843 854 10.1080/00222938700770501

[B7] Schrödl M Neusser TP Towards a phylogeny and evolution of Acochlidia (Mollusca: Gastropoda: Opisthobranchia) Zool J Linn Soc 2010 158 124 154 10.1111/j.1096-3642.2009.00544.x

[B8] Markmann M Tautz D Reverse taxonomy: an approach towards determining the diversity of meiobenthic organisms based on ribosomal RNA signature sequences Philos T R Soc B 2005 360 1462 1917 1924 10.1098/rstb.2005.1723 PMC1609229 16214749

[B9] Hebert PDN Cywinska A Ball SL DeWaard JR Biological identifications through DNA barcodes Proc R Soc Lond B Biol Sci 2003 270 1512 313 321 10.1098/rspb.2002.2218 PMC1691236 12614582

[B10] Vogler AP Monaghan MT Recent advances in DNA taxonomy J Zool Syst Evol Res 2006 45 1 1 10

[B11] Hart MW Sunday J Things fall apart: biological species form unconnected parsimony networks Biol Lett 2007 3 509 512 10.1098/rsbl.2007.0307 17650475 PMC2391196

[B12] Meyer CP Paulay G DNA barcoding: Error rates based on comprehensive sampling PLoS Biol 2005 3 12 2229 2238 10.1371/journal.pbio.0030422 PMC1287506 16336051

[B13] Wiemers M Fiedler K Does the DNA barcoding gap exist? - a case study in blue butterflies (Lepidoptera: Lycaenidae) Front Zool 2007 4 8 10.1186/1742-9994-4-8 17343734 PMC1838910

[B14] Davison A Blackie RLE Scothern GP DNA barcoding of stylommatophoran land snails: a test of existing sequences Mol Ecol Resour 2009 9 4 1092 1101 10.1111/j.1755-0998.2009.02559.x 21564847

[B15] Weigand AM Jochum A Pfenninger M Steinke D Klussmann-Kolb A A new approach to an old conundrum-DNA barcoding sheds new light on phenotypic plasticity and morphological stasis in microsnails (Gastropoda, Pulmonata, Carychiidae) Mol Ecol Resour 2011 11 2 255 265 10.1111/j.1755-0998.2010.02937.x 21429131

[B16] Sauer J Hausdorf B A comparison of DNA-based methods for delimiting species in a Cretan land snail radiation reveals shortcomings of exclusively molecular taxonomy Cladistics 2012 28 3 300 316 10.1111/j.1096-0031.2011.00382.x 34872193

[B17] Meier R Shiyang K Vaidya G Ng PKL DNA barcoding and taxonomy in diptera: A tale of high intraspecific variability and low identification success Syst Biol 2006 55 5 715 728 10.1080/10635150600969864 17060194

[B18] Lohse K Can mtDNA barcodes be used to delimit species? A response to Pons et al Syst Biol 2009 58 4 439 441 10.1093/sysbio/syp039 20525596

[B19] Song H Buhay JE Whiting MF Crandall KA Many species in one: DNA barcoding overestimates the number of species when nuclear mitochondrial pseudogenes are coamplified Proc Natl Acad Sci U S A 2008 105 36 13486 13491 10.1073/pnas.0803076105 18757756 PMC2527351

[B20] Rubinoff D Cameron S Will K A genomic perspective on the shortcomings of mitochondrial DNA for "barcoding" identification J Hered 2006 97 6 581 594 10.1093/jhered/esl036 17135463

[B21] Hausdorf B Hennig C Species delimitation using dominant and codominant multilocus markers Syst Biol 2010 59 5 491 503 10.1093/sysbio/syq039 20693311

[B22] Zhang C Zhang D-X Zhu T Yang Z Evaluation of a Bayesian coalescent method of species delimitation Syst Biol 2011 60 6 747 761 10.1093/sysbio/syr071 21876212

[B23] Barr NB Cook A Elder P Molongoski J Prasher D Robinson DG Application of a DNA barcode using the 16S rRNA gene to diagnose pest Arion species in the USA J Molluscan Stud 2009 75 187 191 10.1093/mollus/eyn047

[B24] Feng YW Li Q Kong LF Zheng XD DNA barcoding and phylogenetic analysis of Pectinidae (Mollusca: Bivalvia) based on mitochondrial COI and 16S rRNA genes Mol Biol Rep 2011 38 1 291 299 10.1007/s11033-010-0107-1 20336381

[B25] Pfenninger M Magnin F Phenotypic evolution and hidden speciation in Candidula unifasciata ssp (Helicellinae, Gastropoda) inferred by 16S variation and quantitative shell traits Mol Ecol 2001 10 10 2541 2554 10.1046/j.0962-1083.2001.01389.x 11742553

[B26] Sonnenberg R Nolte AW Tautz D An evaluation of LSU rDNA D1-D2 sequences for their use in species identification Front Zool 2007 4 6 1 12 17306026 10.1186/1742-9994-4-6 PMC1805435

[B27] Papadopoulou A Monaghan MT Barraclough TG Vogler AP Sampling error does not invalidate the Yule-Coalescent Model for species delimitation. A Response to Lohse (2009) Syst Biol 2009 58 4 442 444 10.1093/sysbio/syp038

[B28] Lim GS Balke M Meier R Determining species boundaries in a world full of rarity: singletons, species delimitation methods Syst Biol 2012 61 1 165 169 10.1093/sysbio/syr030 21482553

[B29] Andrade SCS Norenburg JL Solferini VN Worms without borders: genetic diversity patterns in four Brazilian Ototyphlonemertes species (Nemertea, Hoplonemertea) Mar Biol 2011 158 9 2109 2124 10.1007/s00227-011-1718-3

[B30] Monaghan MT Balke M Gregory TR Vogler AP DNA-based species delineation in tropical beetles using mitochondrial and nuclear markers Philos T R Soc B 2005 360 1462 1925 1933 10.1098/rstb.2005.1724 PMC1609231 16214750

[B31] Monaghan MT Balke M Pons J Vogler AP Beyond barcodes: complex DNA taxonomy of a south pacific island radiation Proc R Soc B-Biol Sci 2006 273 1588 887 893 10.1098/rspb.2005.3391 PMC1560222 16618684

[B32] Monaghan MT Wild R Elliot M Fujisawa T Balke M Inward DJ Lees DC Ranaivosolo R Eggleton P Barraclough TG Accelerated species inventory on Madagascar using coalescent-based models of species delineation Syst Biol 2009 58 3 298 311 10.1093/sysbio/syp027 20525585

[B33] Pons J Barraclough TG Gomez-Zurita J Cardoso A Duran DP Hazell S Kamoun S Sumlin WD Vogler AP Sequence-based species delimitation for the DNA taxonomy of undescribed insects Syst Biol 2006 55 595 609 10.1080/10635150600852011 16967577

[B34] Hendrich L Pons J Ribera I Balke M Mitochondrial cox1 sequence data reliably uncover patterns of insect diversity but suffer from high lineage-idiosyncratic error rates PLoS One 2010 5 12 e14448 10.1371/journal.pone.0014448 21203427 PMC3010977

[B35] Fontaneto D Kaya M Herniou EA Barraclough TG Extreme levels of hidden diversity in microscopic animals (Rotifera) revealed by DNA taxonomy Mol Phylogenet Evol 2009 53 1 182 189 10.1016/j.ympev.2009.04.011 19398026

[B36] Birky CW Ricci C Melone G Fontaneto D Integrating DNA and morphological taxonomy to describe diversity in poorly studied microscopic animals: new species of the genus Abrochtha Bryce, 1910 (Rotifera: Bdelloidea: Philodinavidae) Zool J Linn Soc 2011 161 4 723 734 10.1111/j.1096-3642.2010.00674.x

[B37] Templeton AR Crandall KA Sing CF A cladistic analysis of phenotypic associations with haplotypes inferred from restriction endonuclease mapping and DNA-sequence data III. Cladogram estimation Genetics 1992 132 2 619 633 1385266 10.1093/genetics/132.2.619 PMC1205162

[B38] Yang ZH Rannala B Bayesian species delimitation using multilocus sequence data Proc Natl Acad Sci U S A 2010 107 20 9264 9269 10.1073/pnas.0913022107 20439743 PMC2889046

[B39] Puillandre N Lambert A Brouillet S Achaz G ABGD, Automatic Barcode Gap Discovery for primary species delimitation Mol Ecol 2012 21 8 1864 1877 10.1111/j.1365-294X.2011.05239.x 21883587

[B40] Jörger KM Neusser TP Haszprunar G Schrödl M Undersized and underestimated: 3D-visualization of the Mediterranean interstitial acochlidian gastropod Pontohedyle milaschewitchii (Kowalevsky, 1901) Org Divers Evol 2008 8 194 214 10.1016/j.ode.2007.09.002

[B41] Jörger KM Neusser TP Schrödl M Re-description of a female Pontohedyle brasilensis (Rankin, 1979), a junior synonym of the Mediterranean P. milaschewitchii (Kowalevsky, 1901) (Acochlidia, Gastropoda) Bonner Zool Beiträge 2007 55 3–4 283 290

[B42] Giere O Meiobenthology: The microscopic motile fauna of aquatic sediments 2009 2 Berlin: Springer Verlag

[B43] Swedmark B On the biology of sexual reproduction of the interstitial fauna of marine sand Proceedings of the 15th International Congress of Zoology London 1959 327 329

[B44] Swedmark B The interstitial fauna of marine sand Biol Rev 1964 39 1 42 10.1111/j.1469-185X.1964.tb00948.x

[B45] Kieneke A Arbizu PMM Fontaneto D Spatially structured populations with a low level of cryptic diversity in European marine Gastrotricha Mol Ecol 2012 21 5 1239 1254 10.1111/j.1365-294X.2011.05421.x 22257178

[B46] Casu M Curini-Galletti M Sibling species in interstitial flatworms: a case study using Monocelis lineata (Proseriata: Monocelididae) Mar Biol 2004 145 4 669 679

[B47] Casu M Lai T Sanna D Cossu P Curini-Galletti M An integrative approach to the taxonomy of the pigmented European Pseudomonocelis Meixner, 1943 (Platyhelminthes: Proseriata) Biol J Linn Soc 2009 98 4 907 922 10.1111/j.1095-8312.2009.01316.x

[B48] Leasi F Todaro M Meiofaunal cryptic species revealed by confocal microscopy: the case of Xenotrichula intermedia (Gastrotricha) Mar Biol 2009 156 6 1335 1346 10.1007/s00227-009-1175-4

[B49] Todaro MA Fleeger JW Hu YP Hrincevich AW Foltz DW Are meiofaunal species cosmopolitan? Morphological and molecular analysis of Xenotrichula intermedia (Gastrotricha: Chaetonotida) Mar Biol 1996 125 4 735 742 10.1007/BF00349256

[B50] Schmidt H Westheide W Are the meiofaunal polychaetes Hesionides arenaria and Stygocapitella subterranea true cosmopolitan species? — results of RAPD-PCR investigations Zool Scr 2000 29 1 17 27 10.1046/j.1463-6409.2000.00026.x

[B51] Schmidt H Westheide W Genetic relationships (RAPD-PCR) between geographically separated populations of the "cosmopolitan" interstitial polychaete Hesionides gohari (Hesionidae) and the evolutionary origin of the freshwater species Hesionides riegerorum Biol Bull 1999 196 2 216 226 10.2307/1542567 28296476

[B52] Westheide W Schmidt H Cosmopolitan versus cryptic meiofaunal polychaete species: an approach to a molecular taxonomy Helgol Mar Res 2003 57 1 1 6

[B53] Jörger KM Stöger I Kano Y Fukuda H Knebelsberger T Schrödl M On the origin of Acochlidia and other enigmatic euthyneuran gastropods, with implications for the systematics of Heterobranchia BMC Evol Biol 2010 10 323 10.1186/1471-2148-10-323 20973994 PMC3087543

[B54] Hebert PDN Ratnasingham S de Waard JR Barcoding animal life: cytochrome c oxidase subunit 1 divergences among closely related species Proc R Soc Lond B Biol Sci 2003 270 S96 S99 10.1098/rsbl.2003.0025 PMC1698023 12952648

[B55] Puillandre N Modica MV Zhang Y Sirovich L Boisselier MC Cruaud C Holford M Samadi S Large-scale species delimitation method for hyperdiverse groups Mol Ecol 2012 21 11 2671 2691 10.1111/j.1365-294X.2012.05559.x 22494453

[B56] Austerlitz F David O Schaeffer B Bleakley K Olteanu M Leblois R Veuille M Laredo C DNA barcode analysis: a comparison of phylogenetic and statistical classification methods BMC Bioinformatics 2009 10 14 S10 10.1186/1471-2105-10-S14-S10 19900297 PMC2775147

[B57] Hickerson MJ Meyer CP Moritz C DNA barcoding will often fail to discover new animal species over broad parameter space Syst Biol 2006 55 5 729 739 10.1080/10635150600969898 17060195

[B58] Knowles LL Carstens BC Delimiting species without monophyletic gene trees Syst Biol 2007 56 6 887 895 10.1080/10635150701701091 18027282

[B59] Hebert PDN Stoeckle MY Zemlak TS Francis CM Identification of birds through DNA barcodes PLoS Biol 2004 2 10 1657 1663 10.1371/journal.pbio.0020312 PMC518999 15455034

[B60] DeSalle R Egan MG Siddall M The unholy trinity: taxonomy, species delimitation and DNA barcoding Philos T R Soc B 2005 360 1462 1905 1916 10.1098/rstb.2005.1722 PMC1609226 16214748

[B61] Avise JC Phylogeography: The history and formation of species 2000 Cambridge: Harvard University Press

[B62] Meier R Zhang G Ali F The use of mean instead of smallest interspecific distances exaggerates the size of the "barcoding gap" and leads to misidentification Syst Biol 2008 57 5 809 813 10.1080/10635150802406343 18853366

[B63] Astrin JJ Stuben PE Misof B Wägele JW Gimnich F Raupach MJ Ahrens D Exploring diversity in cryptorhynchine weevils (Coleoptera) using distance-, character- and tree-based species delineation Mol Phylogenet Evol 2012 63 1 1 14 10.1016/j.ympev.2011.11.018 22155423

[B64] Leaché AD Fujita MK Bayesian species delimitation in West African forest geckos (Hemidactylus fasciatus) Proc R Soc B 2010 277 1697 3071 3077 10.1098/rspb.2010.0662 PMC2982061 20519219

[B65] de Queiroz K Species concepts and species delimitation Syst Biol 2007 56 6 879 886 10.1080/10635150701701083 18027281

[B66] de Queiroz K Howard DJ, Berchlor SH The general lineage concept of species, species criteria, and the process of speciation: A conceptual unification and terminological recommendations Endless Forms: Species and Speciation 1998 Oxford, England: Oxford University Press 57 75

[B67] Moritz C Strategies to protect biological diversity and the evolutionary processes that sustain it Syst Biol 2002 51 2 238 254 10.1080/10635150252899752 12028731

[B68] Rankin JJ A freshwater shell-less Mollusc from the Caribbean: structure, biotics and contribution to a new understanding of the Acochlidioidea R Ont Mus Life Sci Contrib 1979 116 1 123

[B69] Kowalevsky A Les Hédylidés, étude anatomique Mem Acad Imperiale Sci StPetersbourg 1901 12 8 1 32

[B70] Marcus E Über Philinoglossacea und Acochlidiacea Kieler Meeresforschungen 1954 10 2 215 223

[B71] Eder B Schrödl M Jörger KM Systematics and redescription of the european meiofaunal slug Microhedyle glandulifera (Kowalevsky, 1901) (Heterobranchia: Acochlidia): evidence from molecules and morphology J Molluscan Stud 2011 77 388 400 10.1093/mollus/eyr030

[B72] Neusser TP Jörger KM Schrödl M Cryptic species in tropic sands - Interactive 3D anatomy, molecular phylogeny and evolution of meiofaunal Pseudunelidae (Gastropoda, Acochlidia) PLoS One 2011 6 8 e23313 10.1371/journal.pone.0023313 21912592 PMC3166138

[B73] Challis DA Hedylopsis cornuta and Microhedyle verrucosa, two new Acochlidiacea (Mollusca: Opisthobranchia) from the Solomon Islands Protectorate Trans R Soc N Z (BiolSci) 1970 12 29 40

[B74] Swedmark B The biology of interstitial Mollusca Symp Zool Soc Lond 1968 22 135 149

[B75] Brenzinger B Neusser TP Jörger KM Schrödl M Integrating 3D microanatomy and molecules: natural history of the Pacific freshwater slug Strubellia Odhner, 1937 (Heterobranchia, Acochlidia) with description of a new species J Molluscan Stud 2011 77 351 374 10.1093/mollus/eyr027

[B76] Neusser TP Fukuda H Jörger KM Kano Y Schrödl M Sacoglossa or Acochlidia? 3D-reconstruction, molecular phylogeny and evolution of Aitengidae (Gastropoda: Heterobranchia) J Molluscan Stud 2011 77 332 350 10.1093/mollus/eyr033

[B77] Neusser TP Heß M Schrödl M Tiny but complex - interactive 3D visualization of the interstitial acochlidian gastropod Pseudunela cornuta (Challis, 1970) Front Zool 2009 6 1 20 10.1186/1742-9994-6-20 19747373 PMC2761907

[B78] Neusser TP Schrödl M Tantulum elegans reloaded: a computer-based 3D-visualization of the anatomy of a Caribbean freshwater acochlidian gastropod Invertebr Biol 2007 126 1 18 39 10.1111/j.1744-7410.2007.00073.x

[B79] Jörger KM Heß M Neusser TP Schrödl M Sex in the beach: spermatophores, dermal insemination and 3D sperm ultrastructure of the aphallic mesopsammic Pontohedyle milaschewitchii (Acochlidia, Opisthobranchia, Gastropoda) Mar Biol 2009 156 6 1159 1170 10.1007/s00227-009-1158-5

[B80] Fontaneto D Iakovenko N Eyres I Kaya M Wyman M Barraclough TG Cryptic diversity in the genus Adineta Hudson & Gosse, 1886 (Rotifera: Bdelloidea: Adinetidae): a DNA taxonomy approach Hydrobiologia 2011 662 1 27 33 10.1007/s10750-010-0481-7

[B81] Derycke S Remerie T Backeljau T Vierstraete A Vanfleteren J Vincx M Moens T Phylogeography of the Rhabditis (Pellioditis) marina species complex: evidence for long-distance dispersal, and for range expansions and restricted gene flow in the northeast Atlantic Mol Ecol 2008 17 14 3306 3322 10.1111/j.1365-294X.2008.03846.x 18573165

[B82] Tulchinsky A Norenburg J Turbeville J Phylogeography of the marine interstitial nemertean Ototyphlonemertes parmula (Nemertea, Hoplonemertea) reveals cryptic diversity and high dispersal potential Mar Biol 2012 159 3 661 674 10.1007/s00227-011-1844-y

[B83] Palmer MA Dispersal of marine meiofauna - a review and conceptual model explaining passive tansport and active recruitment Mar Ecol Prog Ser 1988 48 1 81 91

[B84] Palmer MA Hydrodynamics and structure - interactive effects on meiofaunal dispersal J Exp Mar Biol Ecol 1986 104 1–3 53 68

[B85] Boeckner MJ Sharma J Proctor HC Revisiting the meiofauna paradox: dispersal and colonization of nematodes and other meiofaunal organisms in low- and high-energy environments Hydrobiologia 2009 624 1 91 106 10.1007/s10750-008-9669-5

[B86] Rögl F Palaeogeographic considerations for Mediterranean and Paratethys seaways (Oligocene to Miocene) Ann Naturhistorischen Museums Wien A 1998 99 279 310

[B87] Malaquias MAE Reid DG Tethyan vicariance, relictualism and speciation: evidence from a global molecular phylogeny of the opisthobranch genus Bulla J Biogeogr 2009 36 9 1760 1777 10.1111/j.1365-2699.2009.02118.x

[B88] Krug PJ Patterns of speciation in marine gastropods: a review of the phylogenetic evidence for localized radiations in the sea Am Malacol Bull 2011 29 1–2 169 186

[B89] Schizas NV Coull BC Chandler GT Quattro JM Sympatry of distinct mitochondrial DNA lineages in a copepod inhabiting estuarine creeks in the southeastern USA Mar Biol 2002 140 3 585 594 10.1007/s00227-001-0728-y

[B90] Rocha-Olivares A Fleeger JW Foltz DW Decoupling of molecular and morphological evolution in deep lineages of a meiobenthic harpacticoid copepod Mol Biol Evol 2001 18 6 1088 1102 10.1093/oxfordjournals.molbev.a003880 11371597

[B91] Wawra E Zur Anatomie einiger Acochlidia (Gastropoda, Opisthobranchia) mit einer vorläufigen Revision des Systems und einem Anhang über Platyhedylidae (Opisthobranchia, Ascoglossa) 1987 Wien: Universität Wien

[B92] Schrödl M Techniques for collecting interstitial opisthobranchshttp://www.seaslugforum.net/factsheet.cfm?base=inteextr, Sea Slug Forum 2006

[B93] Drummond A Ashton B Buxton S Cheung M Cooper A Heled J Kearse M Moir R Stones-Havas S Strurrock S Geneious v5.4 2010 http://www.geneious.com

[B94] Altschul SF Gish W Miller W Myers EW Lipman DJ Basic local alignment search tool J Mol Biol 1990 215 3 403 410 2231712 10.1016/S0022-2836(05)80360-2

[B95] Edgar RC MUSCLE: multiple sequence alignment with high accuracy and high throughput Nucleic Acids Res 2004 32 5 1792 1797 10.1093/nar/gkh340 15034147 PMC390337

[B96] Talavera G Castresana J Improvement of phylogenies after removing divergent and ambiguously aligned blocks from protein sequence alignments Syst Biol 2007 56 4 564 577 10.1080/10635150701472164 17654362

[B97] Stamatakis A RAxML-VI-HPC: maximum likelihood-based phylogenetic analyses with thousands of taxa and mixed models Bioinformatics 2006 22 21 2688 2690 10.1093/bioinformatics/btl446 16928733

[B98] Posada D jModelTest: phylogenetic model averaging Mol Biol Evol 2008 25 7 1253 1256 10.1093/molbev/msn083 18397919

[B99] Swofford DL PAUP* Phylogenetic analysis using parsimony (*and other methods) 2002 In. Sunderland MA: Sinauer Associates

[B100] Drummond AJ Rambaut A BEAST: Bayesian evolutionary analysis by sampling trees BMC Evol Biol 2007 7 214 10.1186/1471-2148-7-214 17996036 PMC2247476

[B101] Drummond AJ Ho SYW Phillips MJ Rambaut A Relaxed phylogenetics and dating with confidence PLoS Biol 2006 4 5 699 710 10.1371/journal.pbio.0040088 PMC1395354 16683862

[B102] Clement M Posada D Crandall KA TCS: a computer program to estimate gene genealogies Mol Ecol 2000 9 10 1657 1659 10.1046/j.1365-294x.2000.01020.x 11050560

[B103] Rannala B Yang Z Bayes estimation of species divergence times and ancestral population sizes using DNA sequences from multiple loci Genetics 2003 164 4 1645 1656 12930768 10.1093/genetics/164.4.1645 PMC1462670

