# Supplementary figures and images for: Barcoding against a paradox? Combined molecular species delineations reveal multiple cryptic lineages in elusive meiofaunal sea slugs
Source: BMC Evol Biol. 2012 Dec 18;12:245. doi: 10.1186/1471-2148-12-245 (PMC3573953; doi:10.1186/1471-2148-12-245)

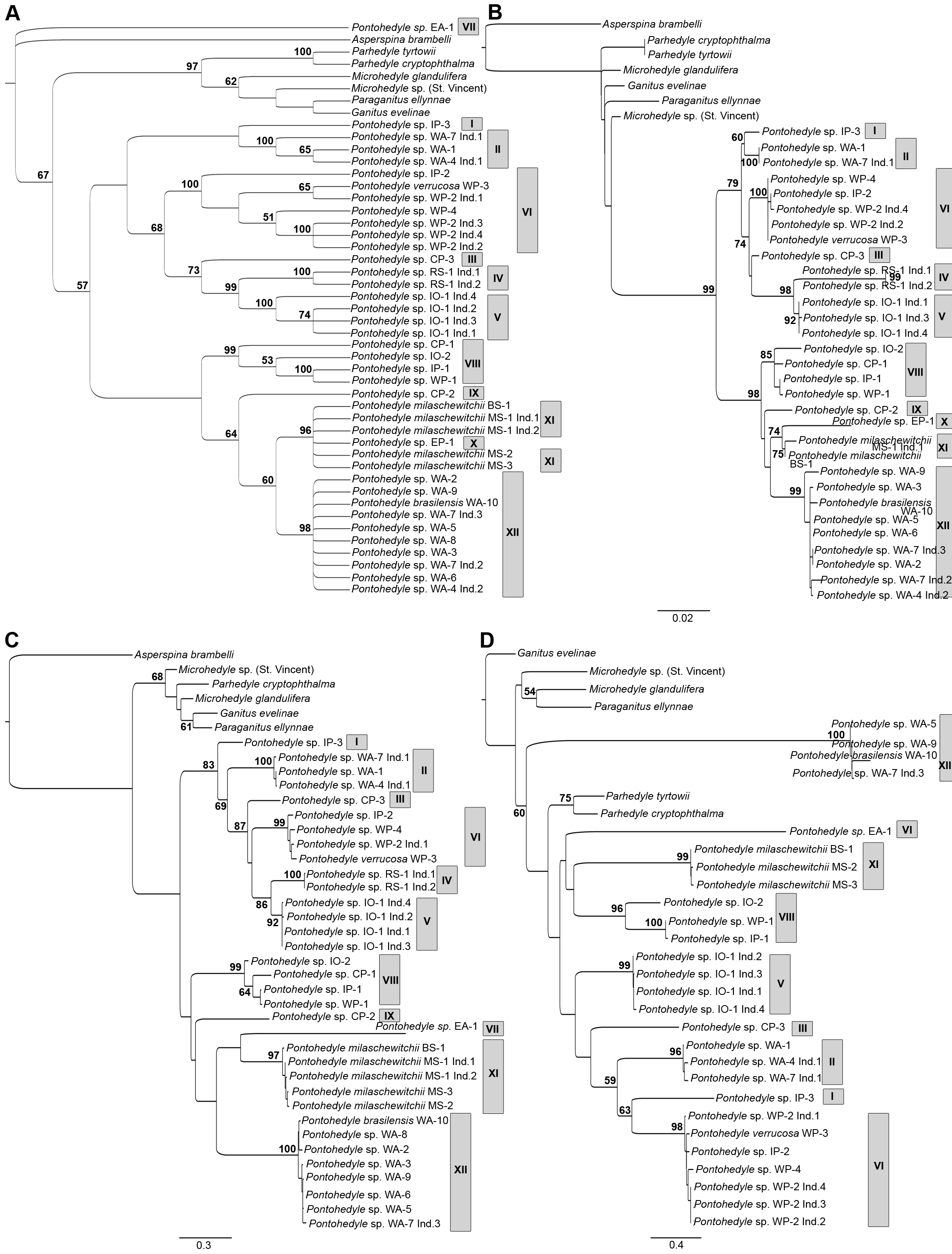

Supplement: Additional file 1 — Additional phylogenetic analyses of the concatenated and single-gene dataset (bootstrap values ≥ 50 given above nodes). A. Maximum parsimony analyses conducted with PAUP on the concatenated three marker dataset. B. Maximum likelihood (ML) single-gene tree of nuclear 28S rRNA. C. ML single-gene tree of mitochondrial 16S rRNA (ambiguous parts in the alignment masked with GBlocks). D. ML single-gene tree of mitochondrial COI (due to extremely long branches Asperspina brambelli was considered as too distant and excluded from the analysis). [file 1471-2148-12-245-S1.tiff]
