# Supplementary material for: Barcoding against a paradox? Combined molecular species delineations reveal multiple cryptic lineages in elusive meiofaunal sea slugs
Source: BMC Evol Biol. 2012 Dec 18;12:245. doi: 10.1186/1471-2148-12-245 (PMC3573953; doi:10.1186/1471-2148-12-245)
Supplement: Additional file 2 — Molecular data analyzed in the present study. Museum numbers (ZSM – Bavarian State Collection of Zoology, SI – Smithsonian Institute (numbers refer to plate coordinates), AM – Australian Museum), DNA vouchers (all at ZSM) and GenBank accession numbers. Sequences retrieved from GenBank are marked with *. [file 1471-2148-12-245-S2.doc]

| Species/ MOTUs | | | Locality | | Museums number | | DNA voucher | | GenBank accession numbers | | | | |
| --- | --- | --- | --- | --- | --- | --- | --- | --- | --- | --- | --- | --- | --- |
|  |  |  |  |  |  |  |  |  | 28S rRNA | | 16S rRNA | | COI |
| *P. milaschewitchii* (MOTU XI) | | | BS-1 | | ZSM Mol 20071381 | | AB34404214 | | JQ410926 | | JQ410925 | | JQ410897 |
|  |  |  | MS-1 | | ZSM Mol 20080054 | | AB34404241 | | JF828043* | | HQ168422* | | - |
|  |  |  | MS-1 | | ZSM Mol 20080055 | | AB34404239 | | - | | JQ410927 | | - |
|  |  |  | MS-2 | | ZSM Mol 20080925 | | - | | - | | JQ410928 | | HQ168459* |
|  |  |  | MS-3 | | ZSM Mol 20080953 | | AB35081832 | | - | | JQ410929 | | JQ410898 |
| *Pontohedyle* sp. 7 (MOTU VII) | | | EA-1 | | ZSM Mol 20071133 | | AB34404268 | | - | | JQ410930 | | JQ410899 |
| *P. brasilensis*  (MOTU XII) | | | WA-10 | | ZSM Mol 20110722 | | AB34402086 | | JQ410932 | | JQ410931 | | JQ410900 |
| *Pontohedyle* sp.2 (MOTU II) | | | WA-1 | | ZSM Mol 20090197 | | AB34858164 | | JQ410934 | | JQ410933 | | JQ410901 |
| *P. brasilensis* (MOTU XII) | | | WA-2 | | ZSM Mol 20090198 | | AB35081813 | | JQ410936 | | JQ410935 | | - |
| *Pontohedyle* sp.2 (MOTU II) | | | WA-4 | | SI-CBC20 10KJ01-D05 | | AB34402049 | | - | | JQ410937 | | JQ410902 |
|  |  |  | WA-7 | | SI-CBC20 10KJ01-C08 | | AB34402065 | | JQ410939 | | JQ410938 | | JQ410903 |
| *P. brasilenis* (MOTU XII) | | | WA-3 | | SI-CBC20 10KJ01-E03 | | AB34500510 | | JQ410941 | | JQ410940 | | - |
|  |  |  | WA-6 | | SI-CBC20 10KJ01-B07 | | AB34402082 | | JQ410943 | | JQ410942 | | - |
|  |  |  | WA-4 | | SI-CBC20 10KJ01-D07 | | AB34500513 | | JQ410944 | | - | | - |
|  |  |  | WA-7 | | SI-CBC20 10KJ01-B09 | | AB34402031 | | JQ410946 | | JQ410945 | | JQ410904 |
|  |  |  | WA-5 | | SI-CBC20 10KJ01-C09 | | AB34500576 | | JQ410948 | | JQ410947 | | JQ410905 |
|  |  |  | WA-8 | | SI-CBC20 10KJ01-A10 | | AB34402026 | | - | | JQ410949 | | - |
|  |  |  | WA-7 | | SI-CBC20 10KJ02-E01 | | AB34402030 | | JQ410950 | | - | | - |
| *P. brasilenis* (MOTU XII) | | | WA-9 | | ZSM Mol 20110723 | | AB34402034 | | JQ410952 | | JQ410951 | | JQ410906 |
| *Pontohedyle* sp. 4 (MOTU IV) | | | RS-1 | | ZSM Mol 20090471 | | AB35081802 | | JQ410954 | | JQ410953 | | - |
|  |  |  |  |  | ZSM Mol 20090472 | | AB35081838 | | JQ410956 | | JQ410955 | | - |
| *Pontohedyle* sp. 6 (MOTU VIII) | | | IO-2 | | ZSM Mol 20100592 | | AB34402021 | | JQ410958 | | JQ410957 | | JQ410907 |
| *Pontohedyle* sp. 5 (MOTU V) | | | IO-1 | | ZSM Mol 20100595 | | AB34402059 | | JQ410960 | | JQ410959 | | JQ410908 |
|  |  |  |  |  | ZSM Mol 20100596 | | AB34402001 | | - | | JQ410961 | | JQ410909 |
|  |  |  |  |  | ZSM Mol 20100597 | | AB34500571 | | JQ410963 | | JQ410962 | | JQ410910 |
|  |  |  |  |  | ZSM Mol 20100603 | | AB34402020 | | JQ410965 | | JQ410964 | | JQ410911 |
| *Pontohedyle* sp. 1 (MOTU I) | | | IP-3 | | ZSM Mol 20081013 | | AB35081769 | | JQ410967 | | JQ410966 | | JQ410912 |
| *Pontohedyle* sp. 6 (MOTU VIII) | | | IP-1 | | ZSM Mol 20081014 | | AB35081827 | | JQ410969 | | JQ410968 | | JQ410913 |
| *P. verrucosa* (MOTU VI) | | | IP-2 | | ZSM Mol 20071135 | | AB34404221 | | JQ410971 | | JQ410970 | | JQ410914 |
| *Pontohedyle* sp. 6 (MOTU VIII) | | | WP-1 | | ZSM Mol 20100379 | | AB34500521 | | JQ410973 | | JQ410972 | | JQ410915 |
| *P. verrucosa* (MOTU VI) | | | WP-2 | | ZSM Mol 20100388 | | AB34500547 | | - | | - | | JQ410916 |
|  |  |  |  |  | ZSM Mol 20100389 | | AB34402044 | | JQ410974 | | - | | JQ410917 |
|  |  |  |  |  | ZSM Mol 20100390 | | AB34402070 | | JQ410975 | | - | | JQ410918 |
|  |  |  |  |  | ZSM Mol 20100391 | | AB34500531 | | - | | JQ410976 | | JQ410919 |
| *P. verrucosa* (MOTU VI) | | | WP-3 | | ZSM Mol 20071820 | | AB34404223 | | JQ410978 | | JQ410977 | | JQ410920 |
|  |  |  | WP-4 | | ZSM Mol 20080176 | | AB34404286 | | JQ410980 | | JQ410979 | | JQ410921 |
| *Pontohedyle* sp. 6 (MOTU VIII) | | | CP-1 | | AM C. 476051.001 | | AB34402037 | | JQ410982 | | JQ410981 | | - |
| *Pontohedyle* sp. 8 (MOTU IX) | | | CP-2 | | AM C. 476054.001 | | AB34402062 | | JQ410984 | | JQ410983 | | - |
| *Pontohedyle* sp. 3 (MOTU III) | | | CP-3 | | AM C. 476062.001 | | AB34500497 | | JQ410986 | | JQ410985 | | JQ410922 |
| *Pontohedyle* sp. 9 (MOTU X) | | | EP-1 | | ZSM Mol 20080565 | | AB34402000 | | JQ410987 | | - | | - |
| **OUTGROUPS** | | | | | | | | | | | | | |
| **Microhedylidae** | | | | | | | | | | | | | |
| *Microhedyle glandulifera* | GenBank | | | ZSM Mol 20081019 | | AB35081799 | | HQ168449* | | HQ168424* | | HQ168461* | |
| *Microhedyle* sp. St. Vincent |  | | | ZSM Mol 20090193 | | AB35081767 | | JQ410989 | | JQ410988 | | JQ410923 | |
| *Parhedyle cryptophthalma* | GenBank | | | ZSM Mol 20100584 | | AB34599403 | | JF828041* | | JF828042* | | JF828033* | |
| *Parhedyle tyrtowii* | GenBank | | | ZSM Mol 20091369 | | AB35081774 | | JF819813* | | - | | JF819818* | |
| **Ganitidae** | | | | | | | | | | | | | |
| *Ganitus evelinae* | | GenBank | | ZSM Mol 20100328 | | AB34404225 | | JF828044* | | JF828045* | | JF828034* | |
| *Paraganitus ellynnae* | | GenBank | | ZSM Mol 20080170 | | AB34404203 | | HQ168448* | | HQ168423* | | HQ168460* | |
| **Asperspinidae** | | | | | | | | | | | | | |
| *Asperspina brambelli* | |  | | ZSM Mol 20100576 | | AB34402042 | | JQ410991 | | JQ410990 | | JQ410924 | |
